# Common intra‐articular knee injections demonstrate a similar recovery trajectory over 60 months: A systematic review and meta‐analysis of 15,418 participants

**DOI:** 10.1002/jeo2.70537

**Published:** 2025-11-14

**Authors:** Henry K. C. Searle, Siddarth Raj, Fatema Dhaif, Samar Hussain, Conrad Harrison, Imran Ahmed, Nick Parsons, Andrew Metcalfe, Jeremy N. Rodrigues, Chetan Khatri

**Affiliations:** ^1^ Warwick Clinical Trials Unit University of Warwick Coventry UK; ^2^ University Hospital Coventry & Warwickshire Coventry UK; ^3^ Stoke Mandeville Hospital Aylesbury UK; ^4^ Nuffield Department of Orthopaedics, Rheumatology and Musculoskeletal Sciences University of Oxford Oxford UK

**Keywords:** biologics, injection, intra‐articular, knee, recovery trajectory

## Abstract

**Purpose:**

To summarise the recovery trajectories of people undergoing intra‐articular knee injections in randomised‐controlled trials (RCTs) for osteoarthritis (OA) using patient‐reported outcome measures over time.

**Methods:**

A systematic review of published RCTs on intra‐articular knee injections with at least 100 participants enrolled and a minimum of 6‐month follow‐up was conducted. MEDLINE, Embase and Cochrane Central Register of Controlled Trials were searched until April 2025. A meta‐analysis of within‐arm response to treatments was calculated as the standardised mean change and 95% confidence interval (CI) at 1, 3, 6, 9, 12, 24 and 60 months by randomly selecting one arm from each study. Subgroup analyses were performed according to treatment received. The primary outcome measure included the Western Ontario and McMaster Universities Osteoarthritis Index (WOMAC). Secondary outcome measures included the visual analogue scale (VAS), Knee Osteoarthritis Outcome Score and International Knee Documentation Centre Score.

**Results:**

This study included 73 RCTs (15,418 participants). Pooled SMC for total WOMAC was −2.59 (95% CI: −4.35 to −0.84) at 1 month, −3.3 (95% CI: −5.10 to −1.50) at 3 months, −2.58 (95% CI: −3.95 to −1.20) at 6 months, −3.19 (95% CI: −7.18 to 0.79) at 9 months, −2.09 (95% CI: −3.50 to −0.67) at 12 months, −0.05 (95% CI: −1.43 to 1.34) at 24 months and 0.04 (95% CI: −1.50 to 1.59) at 60 months. Hyaluronic acid (HA), autologous blood product and steroid arms all showed a similar trend. A similar trend was seen for all subscores. Placebo or physiotherapy showed little improvement, except for in VAS pain for 6 months.

**Conclusion:**

People undergoing HA, autologous blood product and steroid injections demonstrated a consistent pattern in improvement. This may explain why existing meta‐analyses demonstrate inconsistencies in superior treatment. The lack of improvement in placebo or physiotherapy arms suggests regression to the mean is not evident.

**Clinical Trial:**

PROSPERO CRD42023445663.

**Level of Evidence:**

Level I.

**Abbreviations**
ADL: activities of daily living
CI: confidence interval
HA: hyaluronic acid
IKDC: International Knee Documentation Centre
KOOS: Knee injury and Osteoarthritis Outcome Score
NICE: National Institute for Health and Care Excellence
OA: osteoarthritis
PF: physical function
PRISMA: Preferred Reporting Items for Systematic review and Meta‐analysis
PROMs: patient‐reported outcome measures
PRP: platelet rich plasma
QoL: quality of life
RCTs: randomised‐controlled trials
RoB: risk of bias
SD: standard deviation
SMC: standardised mean change
VAS: visual analogue scale
WOMAC: Western Ontario and McMaster Universities Osteoarthritis Index

## INTRODUCTION

The knee is the most common joint affected by osteoarthritis (OA), with knee OA being among the top 10 most common causes of disability across the world [20, 33, 44, 55]. The global prevalence of knee OA is estimated to be 22.9% in individuals aged over 40, and the annual global incidence of knee OA in 2020 was approximately 86.7 million individuals [11]. Worldwide, over 364 million people live with knee OA [31]. As the global population ages, the burden of knee OA is expected to rise, leading to an increased demand for effective management strategies [49].

For some, the knee OA is not advanced another to require arthroplasty, but they still experience significant pain and decreased function [30]. Intra‐articular injections have been proposed as a potential treatment to provide symptomatic relief for this population group [24]. The majority of injections offered include corticosteroids, hyaluronic acid (HA), platelet‐rich plasma (PRP), cell therapies or a combination thereof [9, 48, 51].

However, there is no international consensus in which options should be offered. In the United Kingdom, NICE only recommends the use of intra‐articular corticosteroids, and state that their benefit lasts 2–10 weeks [34]. International guidelines also recommend steroids for short‐term use only, but with no consensus on HA or PRP [2, 40, 41]. However, the ESSKA Consensus recommended the preclinical and clinical evidence to support the use of PRP in those under 80 years with Kelgren–Lawrence grade 0–III OA [28, 29]. There is also no consensus amongst meta‐analyses with some concluding that the superior injection was PRP [32, 45, 48], others HA and PRP [45], HA alone [17] or corticosteroids [17, 22]. Many studies may assume that any improvement in outcome scores over time is due to the intervention rather than the trajectory or clinical course of the condition. The improvement seen in some studies may be due to regression to the mean whereby people recruited to a clinical trial are at their worst at baseline and in time return back to the population mean [12, 23]. This has been seen in other chronic musculoskeletal conditions where people tend to improve over time regardless of treatment received [1, 3, 4, 26, 46, 52, 56]. These studies pooled together all randomised‐controlled trials (RCTs) in the musculoskeletal condition of interest to summarise treatment responses. RCTs are chosen because they are prospective with well‐defined entry points and outcome follow‐up time points. They are therefore a good information source to understand the recovery trajectory and to better differentiate between true treatment effects and the clinical course or trajectory of the condition. Through understanding the recovery trajectory, clinicians and researchers will be able to better interpret studies assessing clinical superiority. It will also help aid clinical decision‐making, manage patient expectations, and plan future trials to optimise treatment strategies for the underserved population with knee OA where arthroplasty is not yet an option.

Therefore, the aim of this systematic review and meta‐analysis was to assess and summarise the trajectories of PROMs over time for people with knee OA who are treated with intra‐articular injections to examine the pattern of recovery trajectories. We did not aim to compare treatments, but rather summarise the change in outcomes after treatment over time. The hypothesis is that responses to the different treatments follow a similar pattern thus reflecting a regression to the mean, and possibly influencing perceived treatment effectiveness.

## METHODS

The review′s protocol was prospectively registered on PROSPERO (CRD42023445663). The preferred reporting items for systematic review and meta‐analysis (PRISMA) guidelines were followed [38].

### Eligibility criteria

The inclusion criteria were RCTs of *n* ≥ 100 participants and minimum follow‐up of 24 weeks. The *n* ≥ 100 was chosen to reduce the influence of small study effects [36]. A minimum 24 weeks follow‐up was included to ensure that both short‐ and medium‐term were available for a study. Participants of interest were anyone with a radiographic diagnosis of knee OA [27] who received an intra‐articular injection (of any therapeutic substance); or an intra‐articular placebo injection (e.g., normal saline); or no injection (e.g., physiotherapy). Outcomes of interest were studies reporting at least one of the chosen multiitem patient‐reported outcome measures (PROMs): Western Ontario and McMaster Universities Osteoarthritis Index (WOMAC); [5] Knee Injury and Osteoarthritis Outcome Score (KOOS); [47] and International Knee Documentation Centre (IKDC) [21]. These PROMs were chosen as they were the most frequently reported PROMs on the initial search prior to conducting the review. Studies that reported validated pain outcome measures in the form of visual analogue scale (VAS) were also included [43].

Studies excluded were: nonrandomised comparative studies; systematic reviews; case reports; commentaries; abstract, conference publications; animal studies; studies with missing data (e.g., where median/mean data were not provided and study authors did not respond to request for data); and open‐label, pilot, feasibility or phase I/II trials.

### Outcome measures

The primary outcome was the WOMAC score (including total, pain, stiffness and physical function [PF], with lower scores meaning better outcome) [5]. This was chosen after an initial scoping review showed this to be the most commonly reported multiitem PROM. The next most frequent were chosen as secondary outcomes: VAS (lower scores mean lower pain), KOOS subscales (including pain, symptoms, activities of daily living [ADL], sport and recreation, and quality of life [QoL]; lower scores mean worse outcome) and IKDC (lower scores mean worse outcome) [21, 43, 47].

### Search strategy

A search strategy (see Supplementary Information) was devised with the aid of a librarian with articles searched from MEDLINE, Embase and Cochrane Central Register of Controlled Trials (CENTRAL) until April 2025. Articles identified were imported onto a citation management software (Rayyan) [37]. Duplicate articles were removed. Abstracts and titles were screened independently by two review authors (H.K.C.S. and F.D.) according to the inclusion criteria. The full‐text articles were independently screened by two authors (C.K. and S.H.). The full‐text articles were also screened for any remaining citations. Any disagreements were discussed with a senior author (J.N.R.). If multiple articles reported on the same study population, data from the article most recently published were utilised and was classified as the ‘main’ article of that study, with data from shorter follow‐up from earlier published data utilised.

### Risk of bias

The risk of bias for all included studies was assessed via the Cochrane risk‐of‐bias (RoB) 2 tool [50]. The risk of bias was independently assessed by two authors (H.K.C.S. and S.R.). Any discrepancies were resolved by a third author (J.N.R.).

### Data extraction

The following data were independently extracted from each study by three authors (F.D., C.K. and S.H.) and verified by another (H.K.C.S.) study title, study location, intervention received, mean age, sex (male or female) and the PROMs of interest.

Means and standard deviations (SD) for the included PROMs were extracted at each time point. If these were not available, the corresponding author was contacted. If the corresponding author was not available or the data were not available from the corresponding author, means were re‐calculated if presented as a change from baseline means. Failing that, data presented graphically were extracted using a ruler [26]. If no SD was reported, standard error, *p*‐values or confidence intervals (CIs) were used to calculate the SD [18]. Failing this, only the means were extracted, and the study was excluded from the meta‐analysis.

### Data analysis

#### Assessing the general response to treatment

Graphs were used to describe and visualise the change in PROMs from baseline at all reported follow‐up time points in all treatment arms to represent people undergoing intra‐articular injections or control.

#### Assessing and summarising overall response to treatment

The variation in size of response was calculated using the standardised mean change (SMC) scores at 1, 3, 6, 9, 12, 24 and 60 months. These were the most frequently reported time‐points and were chosen as potentially helpful time frames for designing future trials. The SMC was calculated by subtracting the follow‐up mean score from the baseline mean score, before dividing this by the study arm′s baseline SD. Estimates of the variance of SMC scores were calculated and described using 95% CIs. This method has been used in previous similar studies assessing recovery trajectories [4, 26, 46]. SMC scores over 0.8 were considered large, 0.5–0.8 moderate and less than 0.5 small [8].

The SMC scores were pooled to summarise the overall response to treatment at each time point using a random effects meta‐analysis model to give an overall SMC score to summarise how all participants in these trials recover. This was done for each outcome: WOMAC, VAS, KOOS and IKDC. As performed in other similar reviews, to calculate an overall, pooled SMC one study arm was randomly extracted [3, 4, 26]. This was done in order to provide an understanding of an overall pattern of recovery trajectory in people undergoing intra‐articular knee injections, and randomly selecting one arm avoids the potential influence of clustering [3].

Subgroup analysis was then performed to calculate SMC for each specific treatment and each primary and secondary outcome. It was decided to utilise all study arms to maximise the data available. The treatments were grouped as: HA, autologous blood products, corticosteroids, adipose tissue product (e.g., amniotic suspension allograft), botox, combination, placebo and physiotherapy. Injections of PRP and other plasma derivatives were grouped together into autologous blood products to maximise the number of studies to strengthen the meta‐analysis. The SMC with 95% CI were summarised in tables for all treatment, with plots drawn of the data for common treatments: HA, autologous blood product, corticosteroid, physio and placebo.

Sensitivity analyses were performed by calculating the SMC of all arms *versus* randomly selecting one arm to ensure that the interpretation of recovery trajectory did not change.

Pearson′s correlation coefficient (*r*) was also calculated to quantify the strength of association between different time points for the primary outcome. This is of benefit for planning future studies using an adaptive study design [39]. A correlation coefficient of 0.4–0.59 was classed as moderate; 0.6–0.79 classed as strong; and 0.8–1 classed a very strong [16].

Analyses were conducted in R (version 2023, Austria) (https://www.r-project.org/).

## RESULTS

**Figure 1:**
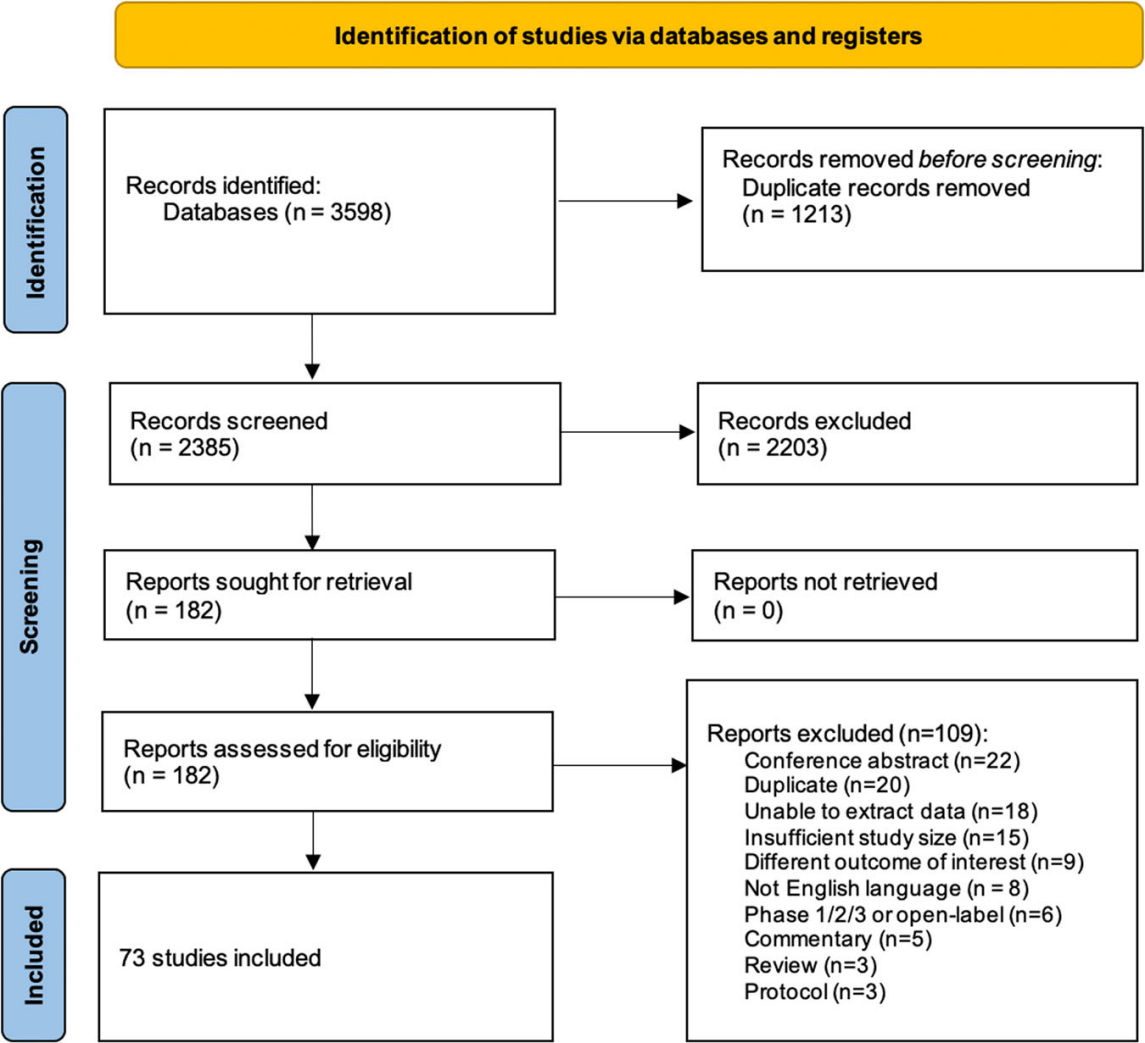
Preferred reporting items for systematic review and meta‐analysis (PRISMA) 2020 flow diagram for included studies.

The search criteria returned 3598 citations. After 1213 duplicates were removed, 2385 citations were screened. One hundred and eighty‐two full‐texts were reviewed and of these 73 studies were included (Figure 1).

### Description of included studies

A summary of the description of studies and their associated references is included in the Supporting Information S1: Table 1. In total this review included 15,418 participants who underwent a trial investigating a knee injectable. Study size ranged from 65 to 1000 participants. The study with 65 participants was included as the number enrolled was 106, but it included a nonsteroidal anti‐inflammatory arm that was not extracted [7]. Mean ages ranged from a 46.2 to 72.0 years.

Sixty‐nine arms reported HA, 37 arms reported autologous blood products, eight arms reported steroid, three arm reported adipose tissue product, three arms reported ozone, four arms reported sprifermin, one arm reported amniotic tissue product, two arms reported botox, seven arms reported combinations of therapy (including PRP + HA, HA + exercise, HA + steroid, HA + steroid), 29 reports reported placebo (saline or dextrose) and seven studies reported physiotherapy.

### Risk of bias assessment

A table summarising the risk of bias assessment can be found at the end of the Supplementary Information Figure S8. Overall, 31/73 (42.5%) were deemed to be a low risk of bias, 31/73 (42.5%) were judged to be of some concern, 11/73 (15.1%) were deemed to be high risk.

## SUMMARY OF TREATMENT RESPONSE

### WOMAC

Thirty‐six studies (85 arms) reported the total score, 35 studies (84 arms) reported the pain score, 29 studies (69 arms) reported the stiffness score; and 32 studies (72 arms) reported PF score.

**Figure 2:**
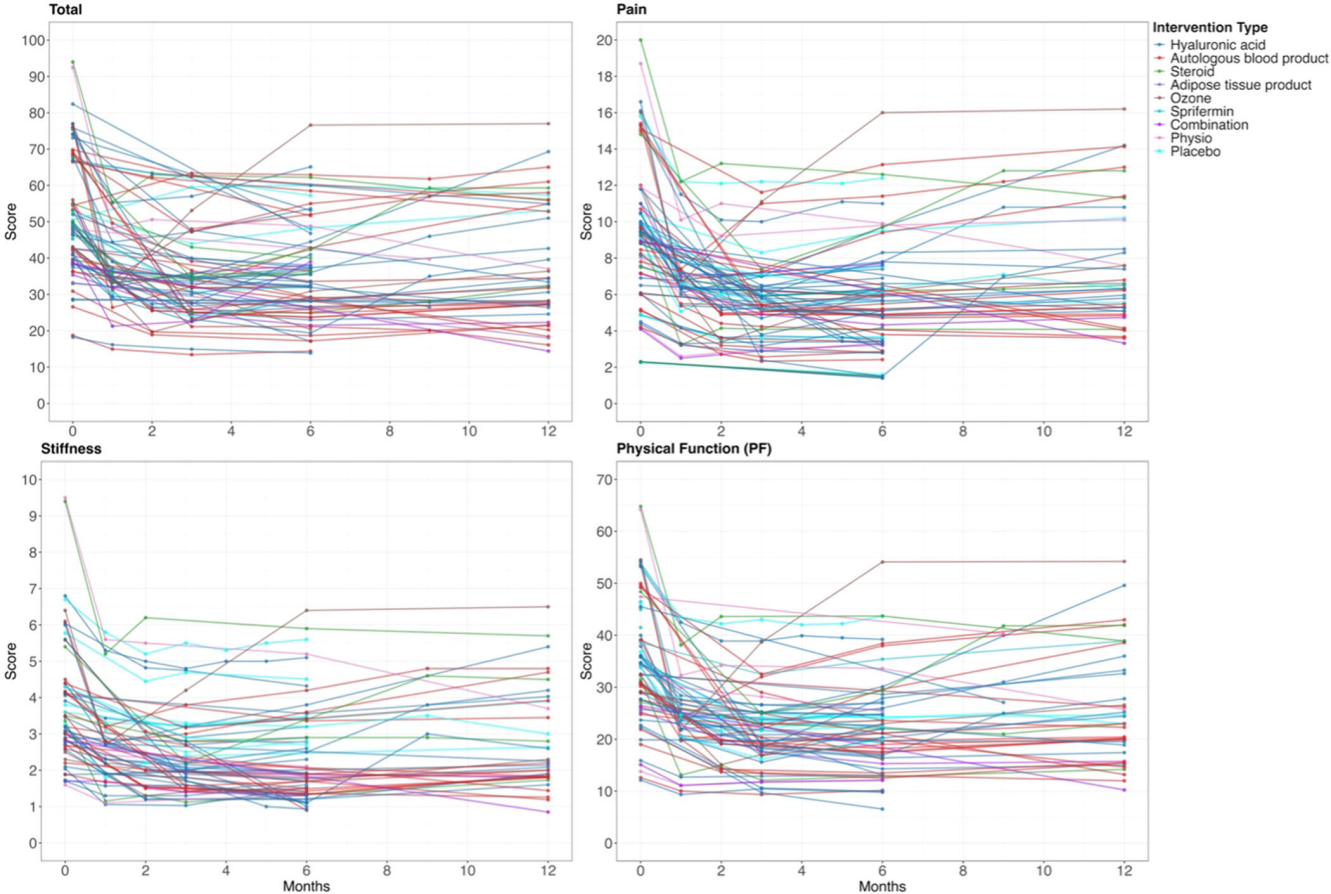
Western Ontario and McMaster Universities Osteoarthritis Index (WOMAC) scores for all intervention types up to 12 months.

A graphical representation of recovery trajectory for each treatment in each arm up to 12 months is shown in Figure 2 and up to 60 months in Supplementary Information, Figures S1–S4. Response lines followed a common pattern of improvement in the first 3–6 months followed by plateau at 6 months.

The pooled WOMAC SMC scores for one randomly selected arm for each trial are displayed in Table 1. This demonstrated the pattern of a large improvement in WOMAC scores in the first 9 months, plateauing at 12 months and returning to baseline at 24 and 60 months. A similar pattern was found when all arms of included trials were included.

**Table 1:** Standardised mean change of Western Ontario and McMaster Universities Osteoarthritis Index (WOMAC) scores for one randomly selected arm.

Time points	Total	Pain	Stiffness	Function
1	−2.59 (−4.35 to −0.84)	−2.35 (−4.08 to −0.62)	−1.12 (−1.84 to −0.41)	−1.53 (−2.22 to −0.84)
3	−3.30 (−5.10 to −1.50)	−2.23 (−3.27 to −1.20)	−1.08 (−1.62 to −0.55)	−1.84 (−2.86 to −0.82)
6	−2.58 (−3.95 to −1.20)	−2.76 (−4.57 to −0.96)	−1.11 (−1.58 to −0.64)	−1.51 (−2.38 to −0.63)
9	−3.19 (−7.18 to 0.79)	−0.39 (−1.52 to 0.74)	−0.76 (−1.90 to 0.37)	−1.90 (−5.16 to 1.37)
12	−2.09 (−3.50 to −0.67)	−1.30 (−2.07 to −0.53)	−0.80 (−1.33 to −0.27)	−1.05 (−1.86 to −0.25)
24	−0.05 (−1.43 to 1.34)	−1.93 (−4.67 to 0.82)	−0.01 (−1.40 to 1.37)	−0.22 (−1.61 to 1.17)
60	0.04 (−1.50 to 1.59)	0.89 (−1.07 to 2.85)	1.22 (−0.75 to 3.18)	0.03 (−1.98 to 2.04)

**Figure 3:**
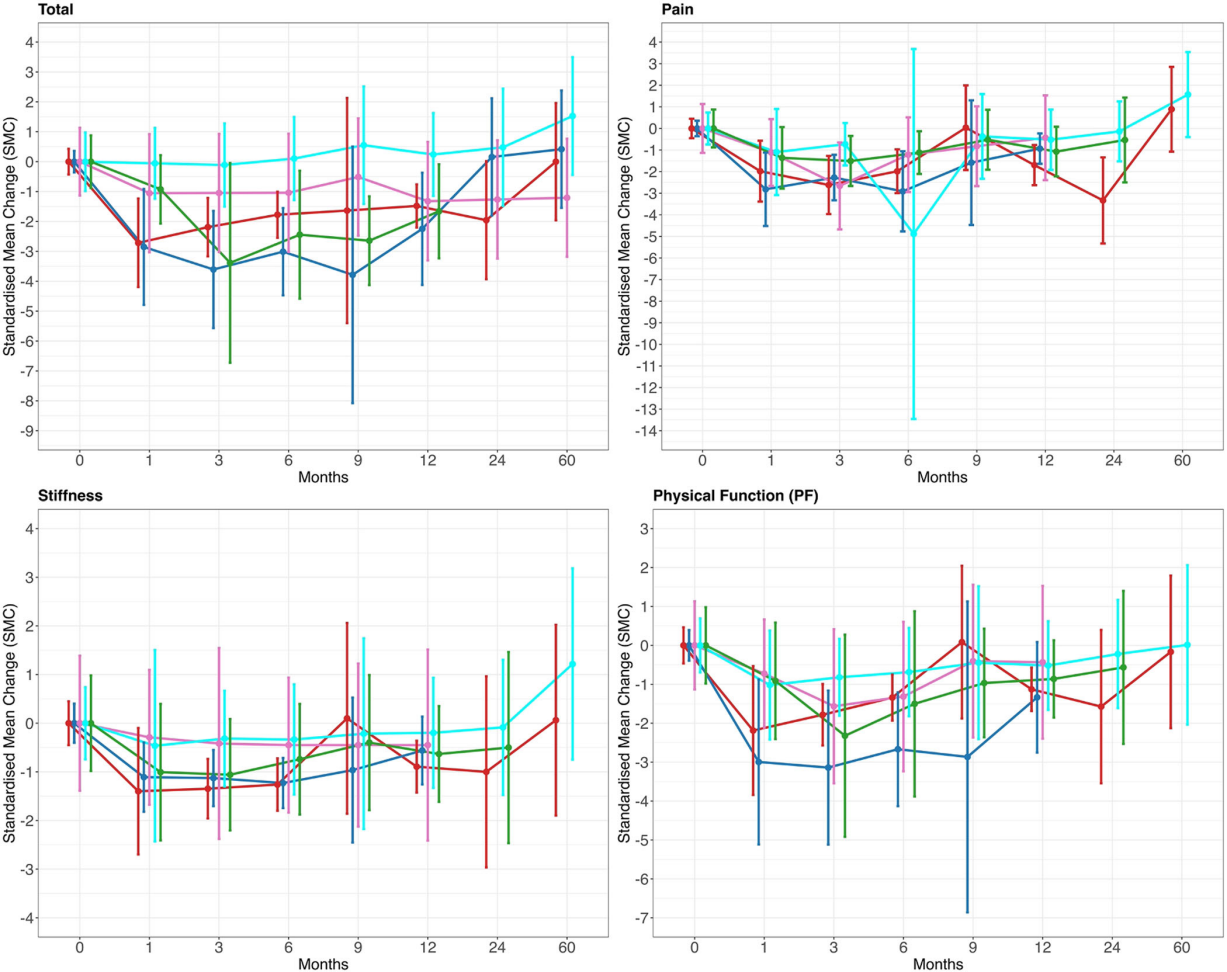
Standardised mean change (SMC) scores for Western Ontario and McMaster Universities Osteoarthritis Index (WOMAC) until 60 months (Blue = hyaluronic acid, red = autologous blood product, green = steroid, pink = physio, cyan = placebo).

The SMC for each treatment is summarised in Supporting Information S1: Tables S2 and S3. The SMC plots for the main treatments are shown in Figure 3. There was an overall similar response in treatments to HA, autologous blood product and steroids. Those receiving placebo, physiotherapy, combination, adipose tissue product or sprifermin had a small effect. No data were reported beyond 24 months for steroid arms. There were no data for amniotic tissue product or botox.

Correlations for the total subscore were overall very strong for each time point (Supporting Information S1: Table S4). For pain scores, correlations were very strong up until 9 months when weaker correlations were seen (Supporting Information S1: Table S5). A similar pattern was seen for stiffness (Supporting Information S1: Table S6) and PF (Supporting Information S1: Table S7).

### VAS

Forty‐nine studies (111 arms) reported VAS. A graphical representation of recovery trajectory for each treatment in each arm up to 24 months is shown in Supporting Information S1: Figure S4. Response line for all treatments showed a rapid early improvement in the 6 months, before plateauing at 24 months.

The pooled VAS SMC scores for one randomly selected arm for each trial were −4.03 (95% CI: −7.31 to −0.74) at 1 month, −3.30 (95% CI: −5.54 to −1.06) at 3 months, −2.93 (−4.63 to −1.23) at 6 months, −1.92 (95% CI: −4.18 to 0.33) at 9 months, −1.69 (95% CI: −2.38 to −1.00) at 12 months, −1.22 (95% CI: −3.53 to 1.09) at 24 months and 0.26 (95% CI: −1.13 to 1.65) at 60 months. A similar pattern was found when all arms of included trials were included.

**Figure 4:**
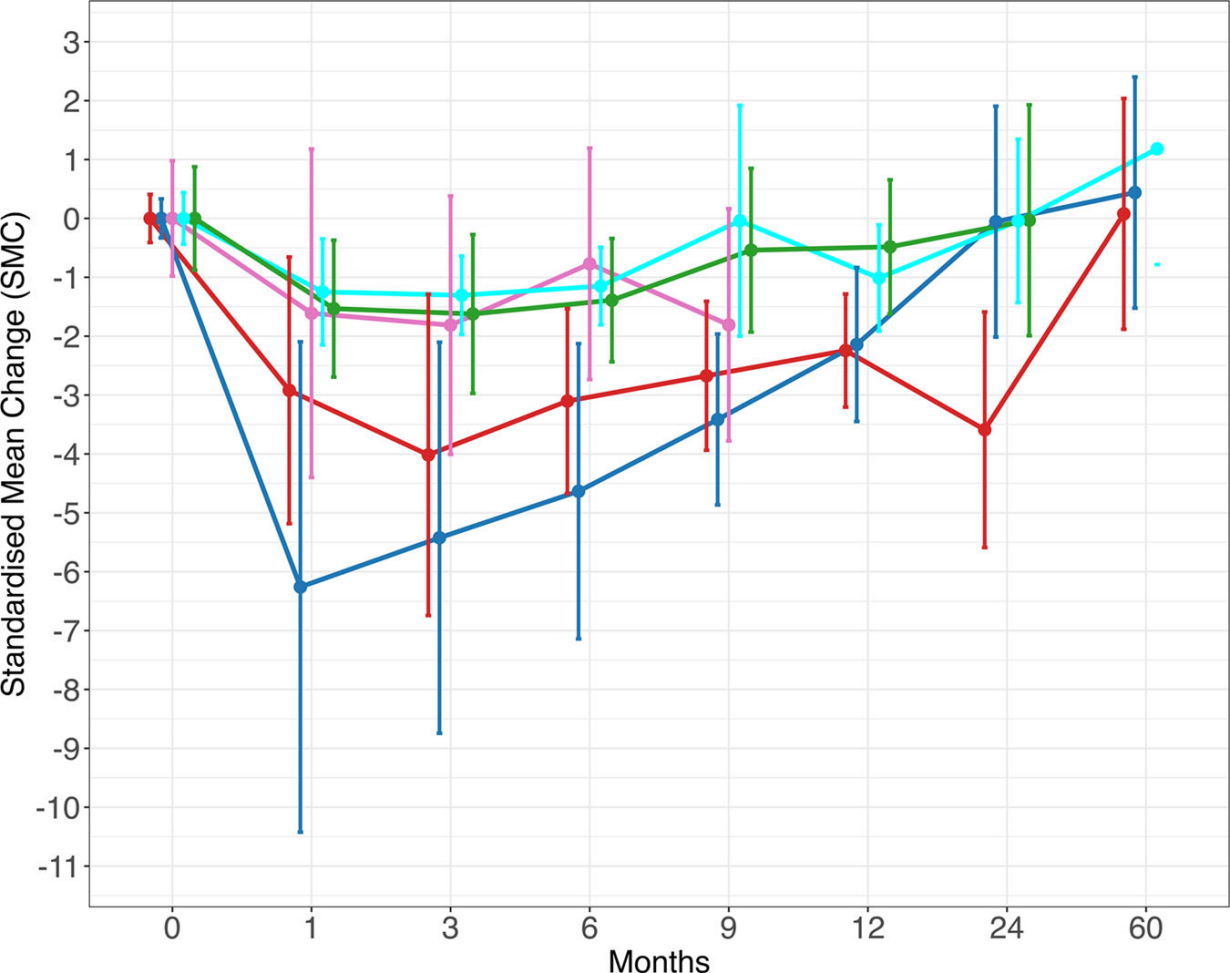
Standardised mean change (SMC) scores for visual analogue scale (VAS) for pain until 60 months (blue = hyaluronic acid, red = autologous blood product, green = steroid, pink = physio, cyan = placebo).

The SMC scores for each treatment are summarised in Supporting Information S1: Tables S8. The SMC for the main treatments are shown in Figure 4. This showed that HA, autologous blood product and steroid showed a similar pattern of improvement. Placebo and physiotherapy had a short‐term effect up to 6 months. Responses for adipose tissue product, ozone, botox and combination varied. There were no data on sprifermin or amniotic tissue product.

### KOOS

Ten studies (23 arms) reported pain, symptoms, sport and recreation. Nine studies (21 arms) reported ADL and QoL. Only studies reporting HA, autologous blood product, adipose tissue product, combination and placebo arms had KOOS data.

Most of the arms reporting KOOS subscales demonstrated an improvement in scores that plateaued after 3 months (Supporting Information S1: Figure S5).

The pooled KOOS SMC scores for one randomly selected arm for each trial all crossed zero are shown in Table 2. This showed a varied pattern of improvement, but results may be limited by the limited number of studies reporting KOOS. This trend was similar when assessing SMC scores for each treatment (Supporting Information S1: Table 9A and 9B, and Figure S6).

**Table 2:** Standardised mean change of knee osteoarthritis outcome scores (KOOS) for one randomly selected arm.

Time points (months)	Pain	Symptoms	Activities of daily living (ADL)	Sport/Recreation	Quality of life (QoL)
1	0.36 (−1.60 to 2.32)	0.13 (1.83 to 2.09)	0.37 (−1.59 to 2.33)	0.16 (−1.80 to 2.12)	0.14 (−1.82 to 2.1)
3	0.54 (−0.45 to 1.52)	0.49 (−0.49 to 1.48)	0.44 (−0.7 to 1.57)	0.45 (−0.53 to 1.43)	0.77 (−0.38 to 1.91)
6	0.52 (−0.22 to 1.26)	0.46 (−0.28 to 1.21)	0.44 (−0.36 to 1.24)	0.51 (−0.24 to 1.25)	0.82 (0.01 to 1.63)
9	0.63 (−1.34 to 2.59)	0.44 (−1.52 to 2.41)	N/A	0.68 (−1.28 to 2.65)	N/A
12	0.67 (−0.07 to 1.42)	0.55 (−0.19 to 1.29)	0.54 (−0.26 to 1.35)	0.59 (−0.15 to 1.33)	0.96 (0.15 to 1.76)

### IKDC

Ten studies (23 arms) reported IKDC. Some arms improved up to 6 months before plateauing and starting to return to baseline scores between 18 and 24 months (Supporting Information S1: Figure S7). Only studies reporting HA, autologous blood product, combination and placebo reported IKDC data.

The pooled IKDC SMC scores for one randomly selected arm showed an inconsistent pattern potentially due to the limited number data available. The SMC was 2.00 (95% CI: −0.02 to 4.02) at 1 month, 0.90 (95% CI: −0.09 to −1.89) at 3 months, 0.96 (0.26 to 1.66) at 6 months, 0.92 (95% CI: −1.06 to 2.89) at 9 months, 0.71 (95% CI: −0.17 to 1.60) at 12 months, 0.03 (95% CI: −1.36 to 1.41) at 24 months and −0.85 (95% CI: −2.82 to 1.11) at 60 months. A similar pattern was found when all arms of included trials were included. An inconsistent pattern was also for each treatment, potentially due to the limited data available (Supporting Information S1: Table S10 and Figure S8).

## DISCUSSION

The key finding of this review is that first, people who undergo HA, autologous blood product or steroids show a similar pattern of improvement in pain, function, QoL, stiffness, ADL scores for the first 9 months before starting to plateau and returning to baseline scores by 24 months. However, the size of response and duration varied. Placebo and physiotherapy arms showed short‐term improvement in VAS pain only for up to 6 months. Other treatment arms had an inconsistent pattern, likely due to the limited data available. Therefore, the hypothesis that all treatments will follow a similar pattern is true for HA, autologous blood product and steroids, but further data are required for other treatments.

The recovery trajectory in this study can be seen to support the use of HA, autologous blood product or steroids, although individual responses may vary including the preparations of injections. The size of response seen in steroids was smaller than HA and autologous blood products. However, in some scores the therapeutic improvement was longer than the 10 weeks NICE states [34]. Although HA had a greater size of response, PRP also demonstrated a treatment effect lasting up to 12 months in keeping with ESSKA Consensus Statement and the United States [28, 29, 35].

Previous reviews have demonstrated a consistent improvement in those receiving placebo in placebo‐controlled trial [42]. It has been hypothesised that this improvement is due to regression to the mean [15]. This review showed that placebo arms experienced a 6‐month improvement in pain scores only, but no improvement in function, stiffness or quality of life. This was also seen in physiotherapy arms. A consistent improvement across all scores would support regression to the mean or a Hawthrone effect, as has been seen in other musculoskeletal conditions, such as rotator cuff tears or meniscal tears [1, 26]. Therefore, there may be some evidence to suggest placebo or physiotherapy can improve pain in 6 months only.

The consistent pattern of improvement for all of the common injections (HA, autologous blood product and steroids) may be explained by the large data and arms available, in comparison to other treatments included (e.g., adipose tissue product, ozone, sprifermin, amniotic tissue product, botox or combination), where the pattern of response was varied. This pattern may also explain why existing reviews have been inconsistent in finding a clinical or statistically superior treatment comparing HA, autologous blood product and steroids. Meta‐analyses and trials report aggregate data rather than individual level data, which can homogenise data [3]. Given the wide heterogeneity in study populations with knee OA, there is a drive to focus on subgroups of patients who may respond differently to certain treatments [6, 19, 54]. As such, one could argue that individual data are better suited, rather than aggregating data as this review has done. However, while individual data will be able to detect individual differences, individual data are often large, not readily available and burdensome, and so aggregate data are often utilised. Aggregating data, as this review has done, is still of benefit for clinicians to use to inform their patients of the general, expected recovery trajectory or clinical course, with knowledge that some individuals may respond differently. It is for future research to identify subgroups of people who may respond better to certain treatments and preparations, and not the aim of this paper which was to compare the pattern of overall recovery trajectories.

Previous studies evaluating the clinical course of OA have focussed on the radiographic progression of OA rather than PROMs assessing health constructs, such as function or pain [25]. Reviews focusing on the latter include longitudinal studies concluding a limited evidence of a decline, but no formal meta‐analysis was performed [10, 53]. When meta‐analysis has been performed on longitudinal studies, heterogeneity was too high to make any reasonable conclusions [13]. Focussing on RCTs alone as in this review allows the inclusion of studies with strict inclusion criteria and well‐defined follow‐up points, which may not be the case for longitudinal, observational studies. Furthermore, including recovery trajectory in RCTs alone will help with the interpretation of existing RCTs and planning of future ones, in particular, the planning of appropriate end‐points of trials using the correlations between scores at different time points. As there is a strong correlation between baseline scores and each follow‐up for WOMAC total and pain scores, researchers could look into adaptive style study design whereby strong correlations across timepoints can be used to strengthen the data in interim analyses [39]. This allows for quicker and less expensive studies, as performed using similar review methodology [39].

### Strengths and limitations

This review provides the first published summary of the recovery trajectory of outcomes for people undergoing intra‐articular knee injections. The review was prospectively registered and performed in accordance with PRISMA guidelines. The inclusion criteria of *n* ≥ 100 participants ensured the avoidance of bias from small study effects [36], and the 6‐month follow‐up ensured we had a mixture of short to medium‐term follow‐up points. Despite strict inclusion criteria, 15,418 participants from 73 studies were included. While other outcome scores are available, the outcomes used in this study were the most frequently reported to maximise the quality of the meta‐analysis. A sensitivity analysis showed that randomly selecting one arm did not reveal any differences in conclusions and avoided the risk of clustering.

Limitations include that, unlike other meta‐analyses, we pooled together different studies with different treatments to assess and present the pattern of recovery trajectory, rather than to calculate the effect size of treatment. For this reason, we also did not calculate effect sizes on differences between treatments. Therefore pooling in this review should be interpreted in the context of this review's aims.

A number of studies (*n* = 18) reported data that could not be utilised for meta‐analysis (e.g., raw mean and SDs, and no reply from study authors), and there may be some bias in the exclusion of these. There were also a fewer number of studies reporting IKDC and KOOS that may limit the interpretations of the recovery trajectory of these outcomes, compared to WOMAC or VAS. There were limited studies on certain, more novel treatments (e.g., adipose tissue product, ozone, sprifermin, amniotic tissue product, botox or combination) and no studies on stem cells. This may be explained by the exclusion of Phase I/II/III studies, and these treatment are known to have a small sample size and short follow‐up periods which may explain the paucity of studies in this review [14].

## CONCLUSION

The consistent pattern of improvement in people undergoing HA, autologous blood product and steroid injections supports their use. However, it may also explain why existing meta‐analyses report differing conclusions in the superior treatment. Given that people undergoing placebo or physiotherapy demonstrate an improvement in VAS pain for 6 months only but no improvement in other scores, suggest that a Hawthorne effect or regression to the mean is not present. The very strong correlations support the use of adaptive style study designs.

## AUTHOR CONTRIBUTIONS

**Henry K. C. Searle**: Conceptualisation; data curation; formal analysis; investigation; methodology; writing—original draft, writing—review and editing. **Siddarth Raj**: Data curation; formal analysis; investigation; methodology; writing—original draft, writing—review and editing. **Fatema Dhaif**: Data curation; investigation; methodology; writing—review and editing. **Samar Hussain**: Data curation; investigation; methodology; writing—review and editing. **Conrad Harrison**: Conceptualisation; formal analysis; investigation; methodology; writing—review and editing. **Imran Ahmed**: Conceptualisation; formal analysis; investigation; methodology; writing—review and editing. **Nick Parsons**: Conceptualisation; formal analysis; supervision; investigation; methodology; writing—review and editing. **Andrew Metcalfe**: Conceptualisation; supervision; investigation; methodology; writing—review and editing. **Jeremy N. Rodrigues**: Conceptualisation; supervision; investigation; methodology; writing—review and editing. **Chetan Khatri**: Conceptualisation; supervision; investigation; methodology; writing—review and editing.

## CONFLICT OF INTEREST STATEMENT

The authors declare no conflict of interest.

## ETHICS STATEMENT

The authors have nothing to report.

## Supporting information

Final Supplementary Information.

## Data Availability

Any further data outwith the data presented in the manuscript and supplementary data are available upon request to the corresponding author.
