# Supplementary material for: Common intra‐articular knee injections demonstrate a similar recovery trajectory over 60 months: A systematic review and meta‐analysis of 15,418 participants
Source: J Exp Orthop. 2025 Nov 14;12(4):e70537. doi: 10.1002/jeo2.70537 (PMC12616398; doi:10.1002/jeo2.70537)
Supplement: Supplementary file 1 — Final Supplementary Information. [file JEO2-12-e70537-s001.docx]

**SEARCH STRATEGY**

**Medline**

1 (knee adj4 (arthritis or osteoarthritis or oa)).mp

2 (intra-artic* adj4 (injection* or injectable* or inject*)).mp.

3 (intraartic* adj4 (injection* or injectable* or inject*)).mp.

4 2 or 3

5 1 and 4

6 limit 5 to randomized controlled trial

7 limit 6 to humans

**Embase**

1 (knee adj4 (arthritis or osteoarthritis or oa)).mp

2 (intra-artic* adj4 (injection* or injectable* or inject*)).mp.

3 (intraartic* adj4 (injection* or injectable* or inject*)).mp.

4 2 or 3

5 1 and 4

6 limit 5 to randomized controlled trial

7 limit 6 to humans

**Cochrane**

1 (knee near/4 (arthritis or osteoarthritis or oa))

2 (intra-artic* near/4 (injection* or injectable* or inject*))

3 (intraartic* near/4 (injection* or injectable* or inject*))

4 #2 or #3

5 #1 and #4

TABLE 1.Demographic of included studies

| **Study ID** | **Country** | **Intervention** | **Control** | **Outcome Measure** | **Number of participants** | | | **Mean age (SD)** | |
| --- | --- | --- | --- | --- | --- | --- | --- | --- | --- |
|  |  |  |  |  | **Total** | **Intervention**  **(M/F)** | **Control**  **(M/F)** | **Intervention** | **Control** |
| **Al-Omran 2014** | Saudi Arabia | Osteonil (HA) | 1. Durolane (HA)  2. Synvisc [hylan G-F 20] (HA) | WOMAC (Total) | 227 | 78 (NR) | 1. 74 (NR)  2. 75 (NR) | NR | NR |
| **Altman 2004** | USA, Canada, Sweden | Durolane (HA) | Saline (Placebo) | WOMAC (Pain, Stiffness, and PF) | 346 | 172 (93/79) | 174 (63/111) | 62.9 (NR) | 63.3 |
| **Altman 1998** | USA  (Multicentre) | Hyalgan (HA) | 1. Saline (Placebo)  2. Naproxen^†^ | VAS | 220 | 105 (41/64) | 1. 115 (54/61)  2. N/A | 62 (10) | 1. 65 (10)  2. N/A |
| **Auw Yang 2008** | Netherlands  (Multicentre) | Autologous conditioned serum - Orthokin (ABP) | Saline (Placebo) | WOMAC (Total)  KOOS (Pain, Symptoms, Sport, QoL^§^, ADL^§^) | 153 | 80 (61/73) | 73 (43/30) | 54 (11) | 53 (11) |
| **Baltzer 2009** | Germany  (Multicentre) | Autologous conditioned serum - Orthokin (ABP) | 1. HYA-Ject (HA)  2. Saline (placebo) | WOMAC (Total^§^, Pain, Stiffness, PF^§^)  VAS | 376 | 134 (69/65) | 1. 135 (61/74)  2. 107 (39/68) | 53.8 (12.2) | 1. 57.4 (12.0)  2. 60.3 (10.7) |
| **Bansal 2021** | India | PRP (ABP) | Monovisc (HA) | WOMAC (Total, Pain, Stiffness and PF) | 132 | 64 (39/25) | 68 (42/26) | 64.4 (NR) | 64.8 (NR) |
| **Barfod 2025** | Denmark | Microfragmented adipose tissue (Adipose tissue product) | Saline (placebo) | KOOS (Pain, Symptoms, ADL, Sports, QoL) | 120 | 60 (24/36) | 60 (26/34) | 52.9 (8.7) | 51.5 (8.9) |
| **Bennell 2021** | Australia  (Multicentre) | PRP (ABP) | Saline (Placebo) | KOOS (Pain, ADL, QoL, Sport, Symptoms)  NRS Pain | 288 | 144 (59/85) | 144 (60/84) | 62.2 (6.3) | 61.6 (6.6) |
| **Berenbaum 2011** | France and Germany | GO-ON (HA) | Hyalgan (HA) | WOMAC^§^ (Pain, Stiffness, PF)  VAS | 426 | 217 (82/135) | 209 (75/134) | 67.2 (7.8) | 66.1 (8.1) |
| **Brandt 2001** | USA  (Multicentre) | Orthovisc (HA) | Saline (Placebo) | WOMAC (Pain, Stiffness, PF) | 226 | 114 (42/72) | 112 (41/71) | 65 (8.4) | 67 (8.4) |
| **Buendía‑López 2018** | Spain | LP-PRP (ABP) | 1. Durolane (HA)  2. NSAID^†^ | WOMAC (Total, Pain, Stiffness, PF) | 65 | 33 (16/17) | 1. 32 (15/17)  2. N/A | 56.15 (3.001) | 1. 56.63 (2.9)  2. N/A |
| **Cerza 2012** | Italy | Autologous condition plasma (ABP) | Hyalgan (HA) | WOMAC (Total) | 120 | 60 (25/35) | 60 (28/32) | 66.5 (11.3) | 66.2 (10.6) |
| **Chen 2020** | USA  (Multicentre) | Synvisc-One [hylan G-F 20] (HA) | Radiofrequency ablation^†^ | WOMAC (Total, Pain^§^, Stiffness^§^ and PF^§^)  NRS Pain | 88 | 88 (34/54) | N/A | 63.1 (9.7) | N/A |
| **Chevalier 2010** | UK, France, Czech Republic, Germany, Belgium and The Netherlands | Synvisc-One [hylan G-F 20] (HA) | Saline (Placebo) | WOMAC (Pain, Stiffness^§^ and PF^§^) | 253 | 124 (32/92) | 129 (41/88) | 63.6 (9.64) | 62.5 (9.17) |
| **Chu 2022** | China  (Multicentre) | LP-PRP (ABP) | Saline (Placebo) | WOMAC (Total, Pain, Stiffness, PF)  VAS | 610 | 308 (123/185) | 302 (127/175) | 53.9 (5.0) | 54.5 (5.1) |
| **Conrozier 2016** | France  (Multicentre) | HAppyVisc® (HA) | Bio-HA - Euflexxa® (HA) | WOMAC (Pain) | 205 | 103 (40/63) | 102 (48/54) | 65.2 (10.1) | 65.3 (10.9) |
| **de Campos 2013** | Brazil | Synvisc-One [hylan G-F 20] & triamcinolone (Combination) | Synvisc-One [hylan G-F 20] (HA) | WOMAC (Total, Pain)  VAS | 104 | 52 (13/39) | 52 (12/40) | 61 (12) | 65 (9) |
| **Dara 2025** | India | LP-PRP (ABP) | Saline (Placebo) | WOMAC (Total)  VAS | 1000 | 500 (192/308) | 500 (188/312) | 58.3 (8.4) | 58.1 (8.7) |
| **Deyle 2020** | USA  (Multicentre) | Triamcinolone acetonide (Steroid) | Physical therapy (Physio) | WOMAC (Total, Pain, Stiffness, PF) | 156 | 78 (40/38) | 78 (41/37) | 56 (8.2) | 56.3 (9.2) |
| **Di Martino 2022** | Italy | LR-PRP (ABP) | LP-PRP (ABP) | IKDC  KOOS (Pain, Symptoms, ADL, Sports, QoL) | 175 | 90 (62/28) | 85 (50/35) | 55.2 (9.8) | 55.7 (10.7) |
| **Di Martino 2018**  **(Additional: Filardo 2015)** | Italy | LR-PRP (ABP) | Hyalubrix (HA) | IKDC | 167 | 85 (53/32) | 82 (47/35) | 52.7 (13.2) | 57.5 (11.7) |
| **Dougados 1993** | France | Hyalectin (HA) | Saline (Placebo) | VAS | 110 | 55 (13/42) | 55 (19/36) | 67.0 (9.7) | 69.0 (10.6) |
| **Duymus 2017** | Turkey | PRP (ABP) | 1. (HA)  2. Ozone | WOMAC (Total, Pain, Stiffness, PF)  VAS | 102 | 33 (1/32) | 1. 34 (1/33)  2. 35 (4/31) | 60.4 (5.1) | 1. 60.3 (9.1)  2. 59.4 (5.7) |
| **Eckstein 2021**  **(Addititonal: Hochberg 2019)** | Argentina, Czech Republic, Denmark, Estonia, Hong Kong, Poland, Romania, USA | Sprifermin 30μg 12-monthly | 1. Sprifermin 30μg 6-monthly  2. Sprifermin 100μg 12-monthly  3. Sprifermin 100μg 6-monthly  4. Saline (Placebo) | WOMAC (Total, Pain^§^, Stiffness^§^, PF) | 519 | 110 (37/73) | 1. 111 (31/80)  2. 110 (33/77)  3. 110 (37/73)  4. 108 (32/76) | 66.5 (NR) | 1. 65.0 (NR)  2. 65.0 (NR)  3. 66.0 (NR)  4. 64.5 (NR) |
| **Elawamy 2021** | Egypt | PRP (ABP) | Radiofrequency ablation^†^ | VAS | 100 | 100 (50/50) | N/A | 47.78 (6.9) | N/A |
| **Farr 2019** | USA  (Multicentre) | Amniotic suspension allograft (Amniotic tissue product) | 1. Monovisc (HA)  2. Saline (Placebo) | KOOS (Pain, Symptoms, ADL, Sports, QoL)  VAS | 200 | 68 (35/33) | 1. 64 (33/31)  2. 68 (37/31) | 55.9 (12.3) | 1. 55.4 (11.0)  2. 54.9 (9.9) |
| **Filardo 2012** | Italy | PRP (ABP) | Hyalubrix (HA) | IKDC  KOOS (Pain, Symptoms, ADL, Sports, QoL) | 109 | 54 (37/17) | 55 (31/24) | 55 (NR) | 58 (NR) |
| **Fossati 2024** | Italy | PRP+HA (Combination) | 1. PRP (ABP)  2. Arthrovisc (HA) | WOMAC (Total, Pain, Stiffness, PF)  KOOS (Pain, Symptoms, ADL, Sports, QoL)  IKDC | 174 | 58 | 1. 58 (/)  2. 58 (/) | 60.7 (10.0) | 1. 60.4 (12.1)  2. 60.7 (11.2) |
| **Görmeli 2017** | Turkey | 3xPRP injections (ABP) | 1. 1xPRP injection & 2xsaline (ABP)  2. 3xOrthovisc injections (HA)  3. Saline (Placebo) | IKDC | 162 | 39 (16/23) | 1. 44 (19/25)  2. 39 (17/22)  3. 40 (20/20) | 53.7 (13.1) | 1. 53.8 (13.4)  2. 53.5 (14.0)  3. 52.8 (12.8) |
| **Guo 2018** | China | Hya-Joint Plus (HA) | Hylan G-F 20 (HA) | WOMAC (Total, Pain, Stiffness, PF)  VAS | 258 | 129 (30/99) | 129 (35/94) | 64.82 (9.23) | 62.02 (11.25) |
| **Housman 2014** | Canada, France, Germany, UK, USA | 2xHyalstan injections (HA) | 1. 1xHyalstan injection (HA)  2. Methylprednisolone acetate (Steroid) | WOMAC (Pain) | 391 | 129 (38/91) | 1. 130 (51/79)  2. 132 (41/91) | 62 (9.7) | 1. 60.6 (9.9)  2. 60.1 (9.3) |
| **Hsieh 2022** | Taiwan | Hyruan & dextrose (HA) | Hyruan & saline (HA) | WOMAC^§^ (Pain, Stiffness, PF)  KOOS (Pain, Symptoms, ADL, Sports, QoL) | 104 | 52 (11/41) | 52 (12/40) | 62.4 (10.4) | 62.8 (9.7) |
| **Huang 2019** | China | PRP (ABP) | 1. Sodium hyaluronate (HA)  2. Corticosterod (Steroid) | WOMAC (Total)  VAS | 120 | 40 (25/15) | 1. 40 (19/21)  2. 40 (21/19) | 54.5 (1.2) | 1. 54.8 (1.1)  2. 54.3 (1.4) |
| **Huang 2011** | Taiwan  (Multicentre) | Hyalgan (HA) | Saline (Placebo) | WOMAC^§^ (Pain, Stiffness, PF)  VAS | 200 | 100 (26/74) | 100 (22/78) | 65.9 (8.1) | 64.2 (8.4) |
| **Huskisson 1999** | UK | Hyalgan (HA) | Saline (Placebo) | VAS | 100 | 50 (12/38) | 50 (21/29) | 65.8 (8.8) | 64.8 (9.3) |
| **Kahan 2003** | France  (Multicentre) | Synvisc-One [hylan G-F 20] (HA) | Conventional treatment (Physio) | WOMAC (Total, Pain^§^, Stiffness^§^, PF)  VAS | 506 | 253 (83/170) | 253 (81/172) | 66 (10) | 66 (10) |
| **Kapoor 2024** | India | Hylan GF 20 (HA) | Conservative management (Physio) | WOMAC (Pain, Stiiffness, PF)  VAS | 248 | 124 (NR) | 124 (NR) | NR | NR |
| **Karlsson 2002** | Sweden  (Multicentre) | Artzal (HA) | 1. Synvisc (HA)  2. Saline (Placebo) | WOMAC (Total, Pain, Stiffness, PF)  VAS | 246 | 92 (30/62) | 1. 88 (31/57)  2. 66 (26/40) | 72 (7) | 1. 70 (7)  2. 71 (6) |
| **Lewis 2022** | Australia | 3xPRP injections (ABP) | 1. 1xPRP & 2xsaline (ABP)  2. Saline (Placebo) | KOOS (Pain, Symptoms, ADL, Sports, QoL)  VAS | 107 | 27 (9/18) | 1. 47 (20/27)  2. 28 (12/16) | 59.4 (8.9) | 1. 55.1 (12.6)  2. 60.1 (9.3) |
| **Li 2024** | China | Artz (HA) + triamcinolone acetate + oral duloxetine (Combination) | Artz (HA) + triamcinolone acetate (Combination) | VAS  WOMAC^§^ (Total, Pain Stiffness, PF) | 75 | NA | 75 (39/36) | NA | 62.3 (7.2) |
| **Lomonte 2015** | Brazil | Triamcinolone hexacetonide(steroid) | Methylprednisolone acetate (Steroid) | WOMAC (Total)  VAS | 100 | 50 (1/49) | 50 (5/45) | 64.8 (8.3) | 66.2 (8.2) |
| **Lundsgaard 2008** | Denmark | Hyalgan (HA) | 1. Saline 20ml (Placebo)  2. Saline 2ml (Placebo) | KOOS (Pain, Symptoms, ADL, Sports, QoL)  VAS | 251 | 84 (36/48) | 1. 83(37/46)  2. 84 (40/44) | 68.8 (6.27) | 1. 69.8 (6.8)  2. 69.6 (7.27) |
| **Maheu 2011** | Belgium, Czech Republic, Estonia, France, Poland | F60027 (HA) | Hylan G-F 20 (HA) | VAS | 130 | 117 (24/93) | 119 (32/87) | 63.8 (6.9) | 63 (6.6) |
| **McAlindon 2017** | USA | Triamcinolone (Steroid) | Saline (Placebo) | WOMAC (Pain, Stiffness, PF)  VAS | 140 | 70 (33/37) | 70 (32/38) | 59.1 (8.3) | 57.2 (7.6) |
| **Migliore 2021** | Belgium, Germany, Hungary, Italy, Poland | High and low MW HA (HA) | Saline (Placebo) | VAS | 692 | 347 (115/232) | 345 (115/230) | 63.7 (8.7) | 63.8 (8.1) |
| **Nunes-Tamashiro 2022** | Brazil | PRP (ABP) | 1. Triamcinolone hexacetonide (Steroid)  2. Saline (Placebo) | WOMAC (Total^§^, Pain, Stiffness, PF) | 100 | 34(4/30) | 1. 33 (3/30)  2. 33 (3/30) | 67.6 (7.4) | 1. 65.8 (6.8)  2. 68.0 (6.2) |
| **Park 2021** | South Korea | PRP (ABP) | Synovian (HA) | WOMAC (Total, Pain, Stiffness, PF)  VAS  IKDC | 110 | 55(16/39) | 55 (8/47) | 60.6 (8.2) | 62.3 (9.6) |
| **Petterson 2019** | USA  (Multicentre) | Monovisc (HA) | Saline (placebo) | WOMAC (Pain^§^, PF)  VAS | 369 | 184 (75/109) | 185 (79/106) | 59.5 (8.0) | 58.7 (9.2) |
| **Raeissadat 2021** | Iran | PRP (ABP) | 1. PRP-derived growth factor (PRP)  2. Hyalgan (HA)  3. Ozone | WOMAC (Total, Pain, Stiffness, PF)  VAS | 200 | 52(13/39) | 1. 51 (14/37)  2. 49 (12/37)  3. 48 (12/36) | 56.09 ( 6.0) | 1. 56.07 (6.3)  2. 57.91 (6.7)  3. 57.60 (6.1) |
| **Raeissadat 2020** | Iran | PRP-derived growth factor (ABP) | Hyalgan (HA) | WOMAC (Total, Pain, Stiffness, PF)  VAS | 102 | 50 (14/36) | 52 (15/37) | 57.08 (7.3) | 58.63 (7.09) |
| **Raeissadat 2018** | Iran | Hyalgan (HA) | Ozone | WOMAC (Total, Pain, Stiffness, PF)  VAS | 141 | 74(18/56) | 67(17/50) | 61.1 (6.3) | 58.1 (6.4) |
| **Raeissadat 2015** | Iran | PRP (ABP) | Hyalgan (HA) | WOMAC (Total, Pain, Stiffness, PF) | 140 | 77 (69/8) | 63 (47/15) | 56.85 (9.13) | 61.13 (7.48) |
| **Raman 2008** | UK | Hylan G-F 20 (HA) | Sodium hyaluronate (HA) | WOMAC (Pain, Stiffness, PF)  VAS | 392 | 199 (NR) | 193 (NR) | NR | NR |
| **Raynauld 2005** | Australia, Canada, USA | Hylan GF-20 single course (HA) | 1. Hylan GF-20 repeat course (HA)  2. Appropriate care (Physio) | WOMAC (Pain, Stiffness^§^, PF^§^) | 254 | 78 (26/52) | 1. 48 (14/34)  2. 128 (35/93) | 63.8 (9.5) | 1. 60.8 (9.2)  2. 63.5 (10.5) |
| **Rezasoltani 2020** | Iran | Physical therapy (physio) | 1. Botox  2. Hyalgan (HA)  3. Dextrose (placebo) | KOOS^§^ (Pain, Symptoms, ADL, Sports, QoL)  VAS | 120 | 30 (12/18) | 1. 30 (8/22)  2. 30 (14/16)  3. 30 (11/19) | 70 (6.3) | 1. 67.7 (7.3)  2. 66.1 (9.1)  3. 64.8 (5.8) |
| **Romandini 2024** | Italy | LR-PRP (ABP) | LP-PRP (ABP) | IKDC  WOMAC (Total, Pain, Stiffness PF)  KOOS (Pain, Symptoms, ADL, Sports, QoL) | 132 | 66 (37/29) | 66 (28/35) | 56.7 (10.2) | 51.9 (13.5) |
| **Saccomanno 2015** | Italy | Orthovisc (HA) | 1. Exercise-based rehab (Physio)  2. HA + Exercise-based rehab (Combination) | WOMAC (Pain, Stiffness, PF) | 159 | 55 (11/42) | 1. 51 (18/33)  2. 53 (15/38) | 62.8 (13.2) | 1. 51.2 (10.1)  2. 61.4 (9.7) |
| **Sdeek 2021** | Egypt | PRP (ABP) | High molecular weight HA (HA) | WOMAC (Total)  IKDC  VAS | 189 | 95 (15/80) | 94 (16/78) | 60.2 (NR) | 59.5 (NR) |
| **Shoma 2021** | Bangladesh | PRP (ABP) | Sylocet (HA) | WOMAC (Pain, Stiffness, PF)  VAS | 133 | 65 (27/38) | 68 (29/39) | 51.3 (6.5) | 52.7 (5.4) |
| **Siddharth 2017** | India | Depomedrol (Steroid) | HA (HA) | WOMAC (Total)  VAS | 150 | 75 (30/45) | 75 (38/37) | 71.4 (1.4) | 69.5 (1.7) |
| **Srikanth 2023** | India | PRP (ABP) | Synvisc-One [hylan G-F 20] (HA) | WOMAC (Total)  VAS | 100 | 50 (40/10) | 50 (41/9) | 49.9 (7.7) | 54.2 (5.4) |
| **Sun 2017** | Taiwan | HYA-Joint Plus (HA) | Synvisc-one (HA) | WOMAC (Total, Pain, Stiffness, PF)  VAS | 121 | 62 (14/48) | 59 (17/42) | 62.7 (8.4) | 62.5 (10.0) |
| **Taftain 2021** | Iran | Hyalgan (HA) | 1. Saline (Placebo)  2. Physiotherapy (Physio) | KOOS^§^ (Pain, Symptoms, ADL, Sports, QoL)  VAS | 100 | 27 (6/21) | 1. 30 (2/28)  2. 43 (6/37) | 61.18 (8.4) | 1. 65.66 (9.7)  2. 62.04 (9.9) |
| **Trueba Davilio 2015** | Spain | Diprospan Hypack® (HA) | Betamethasone (Steroid) | WOMAC (Total, Pain, Stiffness, PF)  VAS | 195 | 97 (38/59) | 98 (41/58) | 62.7 (0.6) | 62.8 (0.6) |
| **van der Weegen 2015** | Netherlands  (Multicentre) | Sodium hyaluronate (HA) | Saline (Placebo) | WOMAC (Total, Pain, Stiffness, PF)  VAS | 196 | 99 (49/50) | 97 (57/47) | 58.7 (9.6) | 60.1 (10.1) |
| **Wang 2022** | Taiwan | PRP (ABP) | HYA-Joint Plus (HA) | WOMAC (Total, Pain, Stiffness, PF) | 110 | 54 (12/42) | 56 (16/40) | 61.87 (5.46) | 63.00 (5.30) |
| **Wang 2018** | Taiwan | HA | HA + betamethasone (Combination) | WOMAC (Total)  VAS | 120 | 60 (14/46) | 60 (16/44) | 63.0 (6.25) | 62.50 (6.55) |
| **Yu 2018** | China | PRP (ABP) | 1. HA  2. PRP+HA (Combination) | WOMAC (Total, Pain, Stiffness, PF) | 288 | 104 (50/54) | 1. 88 (48/40)  2. 96 (50/46) | 46.2 (8.6) | 1. 51.5 (9.3)  2. 46.5 (7.5) |
| **Yurtbay 2022** | Turkey | 1xPRP (PRP) | 1. 1xsaline injection (Placebo)  2. 3xPRP injections (ABP)  3. 3xsaline injection (Placebo) | KOOS^§^ (Pain, Symptoms, ADL, Sports, QoL)  VAS | 237 | 62 (41/21) | 1. 59 (48/11)  2. 63 (54/9)  3. 53 (35/18) | 53.29 (12.97) | 1. 56.29 (10.53)  2. 57.38 (8.78)  3. 53.47 (11.31) |
| **Zaffagnini 2022** | Italy | Microfragmented adipose tissue (Adipose tissue product) | PRP (ABP) | KOOS^§^ (Pain, Symptoms, ADL, Sports, QoL)  IKDC^§^  VAS | 108 | 53 (28/25) | 55 (36/19) | 54.5 (12.1) | 54.1 (10.6) |
| **Zhang 2022** | China | Stromal vascular fraction (Adipose tissue product) | HA | WOMAC (Total)  VAS | 126 | 56 (14/42) | 70 (15/54) | 53.98 (13.69) | 55.63 (12.18) |
| **Zhang 2015** | China  (Multicentre) | Artz (HA) | Durolane (HA) | WOMAC (Pain) | 319 | 158 (31/127) | 161 (42/119) | 60.4 (7.75) | 60.2 (8.06) |
| **Zhuang 2024** | China | 1xPRP dose (ABP) | 1. 3xPRP dose (ABP)  2. 5xPRP dose (ABP) | WOMAC (Total, Pain, Stiffness, PF)  VAS | 106 | 36 (11/25) | 1. 35 (9/26)  2. 35 (10/25) | 58.75 (8.88) | 1. 59.88 (8.34)  2. 59.54 (7.49) |

SD = standard deviation; M/F = male/female

ABP = autologous blood product; ADL = activities of daily living; DMARD = ; HA = hyaluronic acid; IKDC = International Knee Documentation Centre; KOOS = Knee Osteoarthritis and Outcome Score; NA = not applicable; NR = not recorded; PF = Physical Function; PRP = platelet rich plasma; QoL = quality of life; UK = United Kingdom; USA = United States of America; VAS = Visual Analogue Scale; WOMAC = Western Ontario and McMaster Universities Osteoarthritis Index

^†^ = Data not extracted as does not fit inclusion criteria

^§^ = unable to extract data as presented in manuscript (or not raw data i.e. standardised) and unsuccessful in contacting study authors for raw data

**REFERENCES**

1. Al-Omran A, Azam Q. Efficacy of Viscosupplementation in Knee Osteoarthritis : A Clinical Trial of Three Agents. *BMB*. 2014;36(3):150-153. doi:10.12816/0008107

2. Altman RD, Åkermark C, Beaulieu AD, Schnitzer T. Efficacy and safety of a single intra-articular injection of non-animal stabilized hyaluronic acid (NASHA) in patients with osteoarthritis of the knee. *Osteoarthritis Cartilage*. 2004;12(8):642-649.

3. Altman RD, Moskowitz R. Intraarticular sodium hyaluronate (Hyalgan) in the treatment of patients with osteoarthritis of the knee: a randomized clinical trial. Hyalgan Study Group. *J Rheumatol*. 1998;25(11):2203-2212.

4. Auw Yang KG, Raijmakers NJH, Van Arkel ERA, et al. Autologous interleukin-1 receptor antagonist improves function and symptoms in osteoarthritis when compared to placebo in a prospective randomized controlled trial. *Osteoarthritis Cartilage*. 2008;16(4):498-505. doi:10.1016/j.joca.2007.07.008

5. Baltzer AWA, Moser C, Jansen SA, Krauspe R. Autologous conditioned serum (Orthokine) is an effective treatment for knee osteoarthritis. *Osteoarthritis Cartilage*. 2009;17(2):152-160. doi:10.1016/j.joca.2008.06.014

6. Bansal H, Leon J, Pont JL, et al. Platelet-rich plasma (PRP) in osteoarthritis (OA) knee: Correct dose critical for long term clinical efficacy. *Sci Rep*. 2021;11(1):3971. doi:10.1038/s41598-021-83025-2

7. Bennell KL, Paterson KL, Metcalf BR, et al. Effect of Intra-articular Platelet-Rich Plasma vs Placebo Injection on Pain and Medial Tibial Cartilage Volume in Patients With Knee Osteoarthritis: The RESTORE Randomized Clinical Trial. *JAMA*. 2021;326(20):2021-2030. doi:10.1001/jama.2021.19415

8. Berenbaum F, Grifka J, Cazzaniga S, et al. A randomised, double-blind, controlled trial comparing two intra-articular hyaluronic acid preparations differing by their molecular weight in symptomatic knee osteoarthritis. *Ann Rheum Dis*. 2012;71(9):1454-1460. doi:10.1136/annrheumdis-2011-200972

9. Brandt KD, Block JA, Michalski JP, Moreland LW, Caldwell JR, Lavin PT. Efficacy and Safety of Intraarticular Sodium Hyaluronate in Knee Osteoarthritis: *Clin Orthop Relat Res*. 2001;385:130-143. doi:10.1097/00003086-200104000-00021

10. Buendía-López D, Medina-Quirós M, Fernández-Villacañas Marín MÁ. Clinical and radiographic comparison of a single LP-PRP injection, a single hyaluronic acid injection and daily NSAID administration with a 52-week follow-up: a randomized controlled trial. *J Orthop Traumatol*. 2018;19(1):3. doi:10.1186/s10195-018-0501-3

11. Cerza F, Carnì S, Carcangiu A, et al. Comparison Between Hyaluronic Acid and Platelet-Rich Plasma, Intra-articular Infiltration in the Treatment of Gonarthrosis. *Am J Sports Med*. 2012;40(12):2822-2827. doi:10.1177/0363546512461902

12. Chen AF, Khalouf F, Zora K, et al. Cooled Radiofrequency Ablation Compared with a Single Injection of Hyaluronic Acid for Chronic Knee Pain: A Multicenter, Randomized Clinical Trial Demonstrating Greater Efficacy and Equivalent Safety for Cooled Radiofrequency Ablation. *Journal of Bone and Joint Surgery*. 2020;102(17):1501-1510. doi:10.2106/JBJS.19.00935

13. Chevalier X, Jerosch J, Goupille P, et al. Single, intra-articular treatment with 6 ml hylan G-F 20 in patients with symptomatic primary osteoarthritis of the knee: a randomised, multicentre, double-blind, placebo controlled trial. *Ann Rheum Dis*. 2010;69(01):113-119. doi:10.1136/ard.2008.094623

14. Chu J, Duan W, Yu Z, et al. Intra-articular injections of platelet-rich plasma decrease pain and improve functional outcomes than sham saline in patients with knee osteoarthritis. *Knee Surg Sports Traumatol Arthrosc*. 2022;30(12):4063-4071. doi:10.1007/s00167-022-06887-7

15. Conrozier T, Eymard F, Afif N, Balblanc JC, Legré-Boyer V, Chevalier X. Safety and efficacy of intra-articular injections of a combination of hyaluronic acid and mannitol (HAnOX-M) in patients with symptomatic knee osteoarthritis. *Knee*. 2016;23(5):842-848. doi:10.1016/j.knee.2016.05.015

16. de Campos GC, Rezende MU, Pailo AF, Frucchi R, Camargo OP. Adding Triamcinolone Improves Viscosupplementation: A Randomized Clinical Trial. *Clinical Orthopaedics & Related Research*. 2013;471(2):613-620. doi:10.1007/s11999-012-2659-y

17. Deyle GD, Allen CS, Allison SC, et al. Physical Therapy versus Glucocorticoid Injection for Osteoarthritis of the Knee. *N Engl J Med*. 2020;382(15):1420-1429. doi:10.1056/NEJMoa1905877

18. Di Martino A, Boffa A, Andriolo L, et al. Leukocyte-Rich versus Leukocyte-Poor Platelet-Rich Plasma for the Treatment of Knee Osteoarthritis: A Double-Blind Randomized Trial. *Am J Sports Med*. 2022;50(3):609-617. doi:10.1177/03635465211064303

19. Dougados M, Nguyen M, Listrat V, Amor B. High molecular weight sodium hyaluronate (hyalectin) in osteoarth~itis of the knee: a 1 year placebo-controlled trial. *Osteoarthritis Cartilage*. 1993;1:97-103.

20. Duymus TM, Mutlu S, Dernek B, Komur B, Aydogmus S, Kesiktas FN. Choice of intra-articular injection in treatment of knee osteoarthritis: platelet-rich plasma, hyaluronic acid or ozone options. *Knee Surg Sports Traumatol Arthrosc*. 2017;25(2):485-492. doi:10.1007/s00167-016-4110-5

21. Eckstein F, Hochberg MC, Guehring H, et al. Long-term structural and symptomatic effects of intra-articular sprifermin in patients with knee osteoarthritis: 5-year results from the FORWARD study. *Ann Rheum Dis*. 2021;80(8):1062-1069. doi:10.1136/annrheumdis-2020-219181

22. Elawamy. Efficacy of Genicular Nerve Radiofrequency Ablation Versus Intra-Articular Platelet Rich Plasma in Chronic Knee Osteoarthritis: A Single-Blind Randomized Clinical Trial. *Pain Phys*. 2021;24:127-134. doi:10.36076/ppj.2021.24.127-134

23. Farr J, Gomoll AH, Yanke AB, Strauss EJ, Mowry KC, ASA Study Group. A Randomized Controlled Single-Blind Study Demonstrating Superiority of Amniotic Suspension Allograft Injection Over Hyaluronic Acid and Saline Control for Modification of Knee Osteoarthritis Symptoms. *J Knee Surg*. 2019;32(11):1143-1154. doi:10.1055/s-0039-1696672

24. Filardo G, Kon E, Di Martino A, et al. Platelet-rich plasma vs hyaluronic acid to treat knee degenerative pathology: study design and preliminary results of a randomized controlled trial. *BMC Musculoskelet Disord*. 2012;13(1):229. doi:10.1186/1471-2474-13-229

25. Görmeli G, Görmeli CA, Ataoglu B, Çolak C, Aslantürk O, Ertem K. Multiple PRP injections are more effective than single injections and hyaluronic acid in knees with early osteoarthritis: a randomized, double-blind, placebo-controlled trial. *Knee Surg Sports Traumatol Arthrosc*. 2017;25(3):958-965. doi:10.1007/s00167-015-3705-6

26. Guo Y, Yang P, Liu L. Origin and Efficacy of Hyaluronan Injections in Knee Osteoarthritis: Randomized, Double-Blind Trial. *Med Sci Monit*. 2018;24:4728-4737. doi:10.12659/MSM.908797

27. Housman L, Arden N, Schnitzer TJ, et al. Intra-articular hylastan versus steroid for knee osteoarthritis. *Knee Surg Sports Traumatol Arthrosc*. 2014;22(7):1684-1692. doi:10.1007/s00167-013-2438-7

28. Hsieh RL, Lee WC. Effects of Intra-articular Coinjections of Hyaluronic Acid and Hypertonic Dextrose on Knee Osteoarthritis: A Prospective, Randomized, Double-Blind Trial. *Arch Phys Med Rehabil*. 2022;103(8):1505-1514. doi:10.1016/j.apmr.2022.04.001

29. Huang Y, Liu X, Xu X, Liu J. Intra-articular injections of platelet-rich plasma, hyaluronic acid or corticosteroids for knee osteoarthritis: A prospective randomized controlled study. *Orthopäde*. 2019;48(3):239-247. doi:10.1007/s00132-018-03659-5

30. Huang TL, Chang CC, Lee CH, Chen SC, Lai CH, Tsai CL. Intra-articular injections of sodium hyaluronate (Hyalgan®) in osteoarthritis of the knee. a randomized, controlled, double-blind, multicenter trial in the asian population. *BMC Musculoskelet Disord*. 2011;12(1):221. doi:10.1186/1471-2474-12-221

31. Huskisson E. Hyaluronic acid in the treatment of osteoarthritis of the knee. *Rheumatology*. 1999;38(7):602-607. doi:10.1093/rheumatology/38.7.602

32. Kahan A, Lleu PL, Salin L. Prospective randomized study comparing the medicoeconomic benefits of Hylan GF-20 vs. conventional treatment in knee osteoarthritis. *Joint Bone Spine*. 2003;70(4):276-281. doi:10.1016/S1297-319X(03)00043-5

33. Karlsson J. Comparison of two hyaluronan drugs and placebo in patients with knee osteoarthritis. A controlled, randomized, double-blind, parallel-design multicentre study. *Rheumatology*. 2002;41(11):1240-1248. doi:10.1093/rheumatology/41.11.1240

34. Lewis E, Merghani K, Robertson I, et al. The effectiveness of leucocyte-poor platelet-rich plasma injections on symptomatic early osteoarthritis of the knee: the PEAK randomized controlled trial. *Bone Joint J*. 2022;104-B(6):663-671. doi:10.1302/0301-620X.104B6.BJJ-2021-1109.R2

35. Lomonte ABV, De Morais MGV, De Carvalho LO, Zerbini CADF. Efficacy of Triamcinolone Hexacetonide versus Methylprednisolone Acetate Intraarticular Injections in Knee Osteoarthritis: A Randomized, Double-blinded, 24-week Study. *J Rheumatol*. 2015;42(9):1677-1684. doi:10.3899/jrheum.150297

36. Lundsgaard C, Dufour N, Fallentin E, Winkel P, Gluud C. Intra‐articular sodium hyaluronate 2 mL versus physiological saline 20 mL versus physiological saline 2 mL for painful knee osteoarthritis: a randomized clinical trial. *Scandinavian Journal of Rheumatology*. 2008;37(2):142-150. doi:10.1080/03009740701813103

37. Maheu E, Zaim M, Appelboom T, et al. Comparative efficacy and safety of two different molecular weight (MW) hyaluronans F60027 and Hylan G-F20 in symptomatic osteoarthritis of the knee (KOA). Results of a non inferiority, prospective, randomized, controlled trial. *Clin Exp Rheumatol*. 2011;29(3):527-535.

38. McAlindon TE, LaValley MP, Harvey WF, et al. Effect of Intra-articular Triamcinolone vs Saline on Knee Cartilage Volume and Pain in Patients With Knee Osteoarthritis: A Randomized Clinical Trial. *JAMA*. 2017;317(19):1967. doi:10.1001/jama.2017.5283

39. Migliore A, Blicharski T, Plebanski R, et al. Knee Osteoarthritis Pain Management with an Innovative High and Low Molecular Weight Hyaluronic Acid Formulation (HA-HL): A Randomized Clinical Trial. *Rheumatol Ther*. 2021;8(4):1617-1636. doi:10.1007/s40744-021-00363-3

40. Nunes-Tamashiro JC, Natour J, Ramuth FM, et al. Intra-articular injection with platelet-rich plasma compared to triamcinolone hexacetonide or saline solution in knee osteoarthritis: A double blinded randomized controlled trial with one year follow-up. *Clin Rehabil*. 2022;36(7):900-915. doi:10.1177/02692155221090407

41. Park YB, Kim JH, Ha CW, Lee DH. Clinical Efficacy of Platelet-Rich Plasma Injection and Its Association With Growth Factors in the Treatment of Mild to Moderate Knee Osteoarthritis: A Randomized Double-Blind Controlled Clinical Trial As Compared With Hyaluronic Acid. *Am J Sports Med*. 2021;49(2):487-496. doi:10.1177/0363546520986867

42. Petterson SC, Plancher KD. Single intra-articular injection of lightly cross-linked hyaluronic acid reduces knee pain in symptomatic knee osteoarthritis: a multicenter, double-blind, randomized, placebo-controlled trial. *Knee Surg Sports Traumatol Arthrosc*. 2019;27(6):1992-2002. doi:10.1007/s00167-018-5114-0

43. Raeissadat SA, Ghazi Hosseini P, Bahrami MH, et al. The comparison effects of intra-articular injection of Platelet Rich Plasma (PRP), Plasma Rich in Growth Factor (PRGF), Hyaluronic Acid (HA), and ozone in knee osteoarthritis; a one year randomized clinical trial. *BMC Musculoskelet Disord*. 2021;22(1):134. doi:10.1186/s12891-021-04017-x

44. Raeissadat SA, Gharooee Ahangar A, Rayegani SM, Minator Sajjadi M, Ebrahimpour A, Yavari P. Platelet-Rich Plasma-Derived Growth Factor vs Hyaluronic Acid Injection in the Individuals with Knee Osteoarthritis: A One Year Randomized Clinical Trial. *JPR*. 2020;Volume 13:1699-1711. doi:10.2147/JPR.S210715

45. Raeissadat SA, Rayegani SM, Forogh B, Hassan Abadi P, Moridnia M, Rahimi-Dehgolan S. Intra-articular ozone or hyaluronic acid injection: Which one is superior in patients with knee osteoarthritis? A 6-month randomized clinical trial. *JPR*. 2018;Volume 11:111-117. doi:10.2147/JPR.S142755

46. Raeissadat SA, Rayegani SM, Hassanabadi H, et al. Knee Osteoarthritis Injection Choices: Platelet- Rich Plasma (PRP) versus Hyaluronic Acid (A one-year randomized clinical trial). *Clinical Medicine Insights: Arthritis and Musculoskeletal Disorders*. 2015;8:1-8. doi:10.4137/CMAMD.S17894

47. Raman R, Dutta A, Day N, Sharma HK, Shaw CJ, Johnson GV. Efficacy of Hylan G-F 20 and Sodium Hyaluronate in the treatment of osteoarthritis of the knee — A prospective randomized clinical trial. *The Knee*. 2008;15(4):318-324. doi:10.1016/j.knee.2008.02.012

48. Raynauld JP, Goldsmith CH, Bellamy N, et al. Effectiveness and safety of repeat courses of hylan G-F 20 in patients with knee osteoarthritis. *Osteoarthritis Cartilage*. 2005;13(2):111-119. doi:10.1016/j.joca.2004.10.018

49. Rezasoltani Z, Azizi S, Najafi S, Sanati E, Dadarkhah A, Abdorrazaghi F. Physical therapy, intra-articular dextrose prolotherapy, botulinum neurotoxin, and hyaluronic acid for knee osteoarthritis: randomized clinical trial. *International Journal of Rehabilitation Research*. 2020;43(3):219-227.

50. Saccomanno MF, Donati F, Careri S, Bartoli M, Severini G, Milano G. Efficacy of intra-articular hyaluronic acid injections and exercise-based rehabilitation programme, administered as isolated or integrated therapeutic regimens for the treatment of knee osteoarthritis. *Knee Surg Sports Traumatol Arthrosc*. 2016;24(5):1686-1694. doi:10.1007/s00167-015-3917-9

51. Sdeek M, Sabry D, El-Sdeek H, Darweash A. Intra-articular injection of Platelet rich plasma versus Hyaluronic acid for moderate knee osteoarthritis. A prospective, double-blind randomized controlled trial on 189 patients with follow-up for three years. *Acta Orthop Belg*. 2021;87(4):729-734. doi:10.52628/87.4.18

52. Shoma FK, Chowdhury ZR, Hossain F, Mohammad Salek AK, Khasru MR, Khandaker MN. Intra-articular Injection of Hyaluronic Acid (HA) and Plateletrich Plasma (PRP) in the Treatment of Mild and Moderate Osteoarthritis of Knee. *Bangladesh Med Res Counc Bull*. 2022;47(1):62-69. doi:10.3329/bmrcb.v47i1.55800

53. Siddharth R, Harleen U. A Prospective, Randomized Trial on Comparative Study of Intrarticular Hyaluronic Acid with Corticosteroid Injections for the Treatment of Osteoarthritis of the Knee Joint. *Ind Jour of Publ Health Rese & Develop*. 2017;8(2):14. doi:10.5958/0976-5506.2017.00074.2

54. Srikanth P, Shaik MR, Jagarlamudi D, Abhishek V. Comparative Evaluation of Efficacy of Intra-Articular Injection of Platelet Rich Plasma Versus Hyaluronic Acid in Treatment of Early Osteoarthritis of Knee. *International Journal of Pharmaceutical and Clinical Research*. 2023;15(7):93-99.

55. Taftian E, Azizi S, Dadarkhah A, et al. A Single-blind Randomised Trial of Intra-Articular Hyaluronic Acid, Hypertonic Saline, and Physiotherapy in Knee Osteoarthritis. *Muscle Ligaments and Tendons J*. 2021;11(03):416. doi:10.32098/mltj.03.2021.05

56. Trueba Davalillo CÁ, Trueba Vasavilbaso C, Navarrete Álvarez JM, Coronel Granado MP, García Jiménez OA, Gil Orbezo F. Clinical efficacy of intra-articular injections in knee osteoarthritis: a prospective randomized study comparing hyaluronic acid and betamethasone. *OARRR*. 2015;7:9-18. doi:10.2147/OARRR.S74553

57. van der Weegen W, Wullems JA, Bos E, Noten H, Van Drumpt RAM. No Difference Between Intra-Articular Injection of Hyaluronic Acid and Placebo for Mild to Moderate Knee Osteoarthritis: A Randomized, Controlled, Double-Blind Trial. *Arthroplasty*. 2015;30(5):754-757. doi:10.1016/j.arth.2014.12.012

58. Wang YC, Lee CL, Chen YJ, et al. Comparing the Efficacy of Intra-Articular Single Platelet-Rich Plasma(PRP) versus Novel Crosslinked Hyaluronic Acid for Early-Stage Knee Osteoarthritis: A Prospective, Double-Blind, Randomized Controlled Trial. *Medicina*. 2022;58(8):1028. doi:10.3390/medicina58081028

59. Wang S, Wu D, Chang Q, Guo Y, Wang C, Fan W. Intra‑articular, single‑shot co‑injection of hyaluronic acid and corticosteroids in knee osteoarthritis: A randomized controlled trial. *Exp Ther Med*. Published online 2018. doi:10.3892/etm.2018.6371

60. Yu W, Xu P, Huang G, Liu L. Clinical therapy of hyaluronic acid combined with platelet‑rich plasma for the treatment of knee osteoarthritis. *Exp Ther Med*. 2018;16:2119-21125. doi:10.3892/etm.2018.6412

61. Yurtbay A, Say F, Çinka H, Ersoy A. Multiple platelet-rich plasma injections are superior to single PRP injections or saline in osteoarthritis of the knee: the 2-year results of a randomized, double-blind, placebo-controlled clinical trial. *Arch Orthop Trauma Surg*. 2021;142(10):2755-2768. doi:10.1007/s00402-021-04230-2

62. Zaffagnini S, Andriolo L, Boffa A, et al. Microfragmented Adipose Tissue Versus Platelet-Rich Plasma for the Treatment of Knee Osteoarthritis: A Prospective Randomized Controlled Trial at 2-Year Follow-up. *Am J Sports Med*. 2022;50(11):2881-2892. doi:10.1177/03635465221115821

63. Zhang S, Xu H, He B, et al. Mid-term prognosis of the stromal vascular fraction for knee osteoarthritis: a minimum 5-year follow-up study. *Stem Cell Res Ther*. 2022;13(1):105. doi:10.1186/s13287-022-02788-1

64. Zhang H, Zhang K, Zhang X, et al. Comparison of two hyaluronic acid formulations for safety and efficacy (CHASE) study in knee osteoarthritis: a multicenter, randomized, double-blind, 26-week non-inferiority trial comparing Durolane to Artz. *Arthritis Res Ther*. 2015;17(1):51. doi:10.1186/s13075-015-0557-x

65. Zhuang W, Li T, Li Y, et al. The varying clinical effectiveness of single, three and five intraarticular injections of platelet-rich plasma in knee osteoarthritis. *J Orthop Surg Res*. 2024;19(1):284. doi:10.1186/s13018-024-04736-6

66. Hochberg MC, Guermazi A, Guehring H, et al. Effect of Intra-Articular Sprifermin vs Placebo on Femorotibial Joint Cartilage Thickness in Patients With Osteoarthritis: The FORWARD Randomized Clinical Trial. *JAMA*. 2019;322(14):1360. doi:10.1001/jama.2019.14735

67. Filardo G, Di Matteo B, Di Martino A, et al. Platelet-Rich Plasma Intra-articular Knee Injections Show No Superiority Versus Viscosupplementation: A Randomized Controlled Trial. *Am J Sports Med*. 2015;43(7):1575-1582. doi:10.1177/0363546515582027

68. Di Martino A, Di Matteo B, Papio T, et al. Platelet-Rich Plasma Versus Hyaluronic Acid Injections for the Treatment of Knee Osteoarthritis: Results at 5 Years of a Double-Blind, Randomized Controlled Trial. *Am J Sports Med*. 2019;47(2):347-354. doi:10.1177/0363546518814532

69. Sun SF, Hsu CW, Lin HS, Liou IH, Chen YH, Hung CL. Comparison of Single Intra-Articular Injection of Novel Hyaluronan (HYA-JOINT Plus) with Synvisc-One for Knee Osteoarthritis: A Randomized, Controlled, Double-Blind Trial of Efficacy and Safety. *Journal of Bone and Joint Surgery*. 2017;99(6):462-471. doi:10.2106/JBJS.16.00469


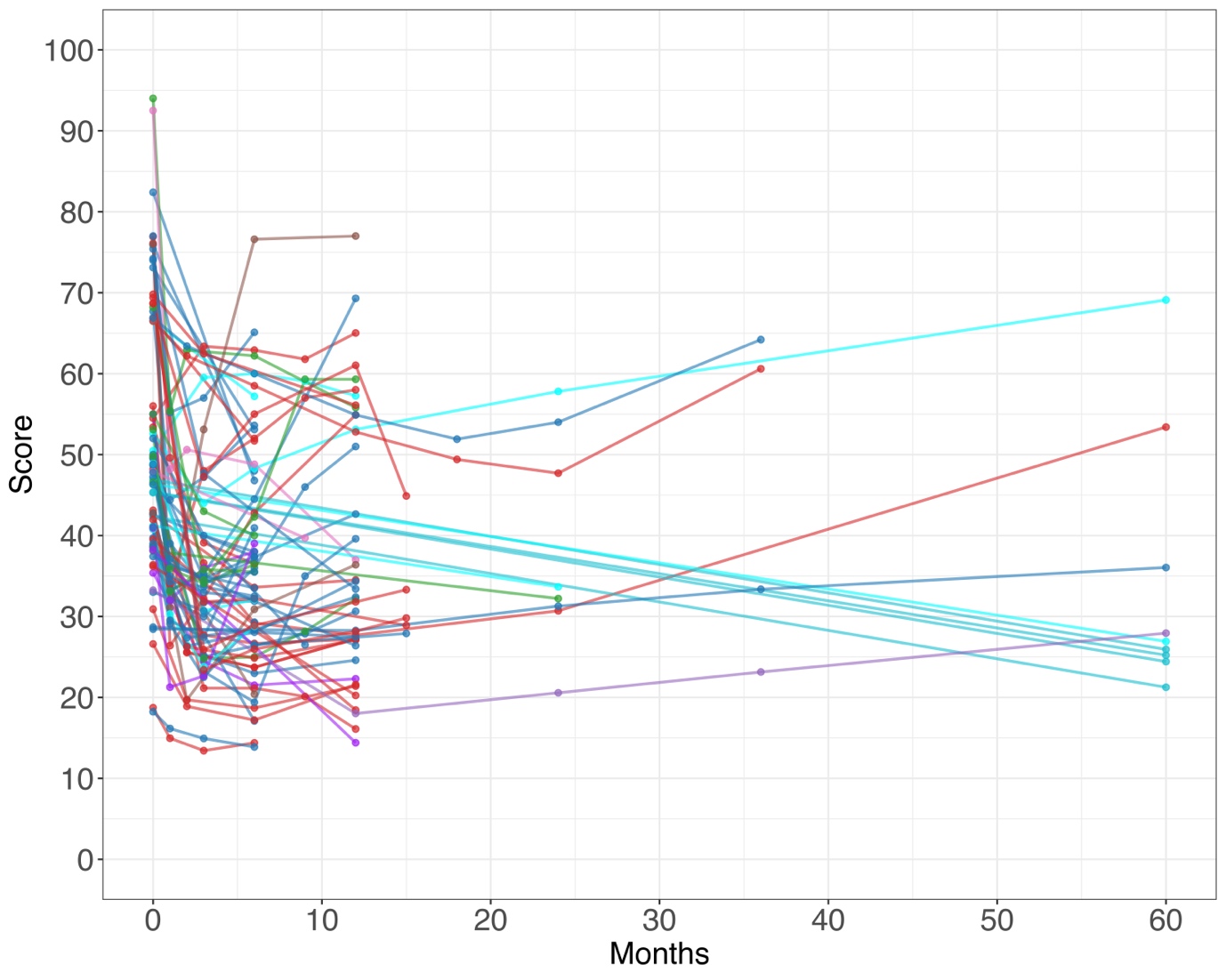

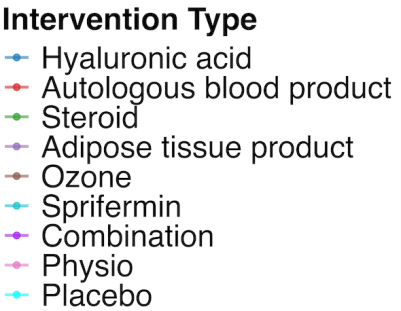


**Fig. 1.** Western Ontario and McMaster Universities Osteoarthritis Index (WOMAC) Total separated by treatment type up to 60 months


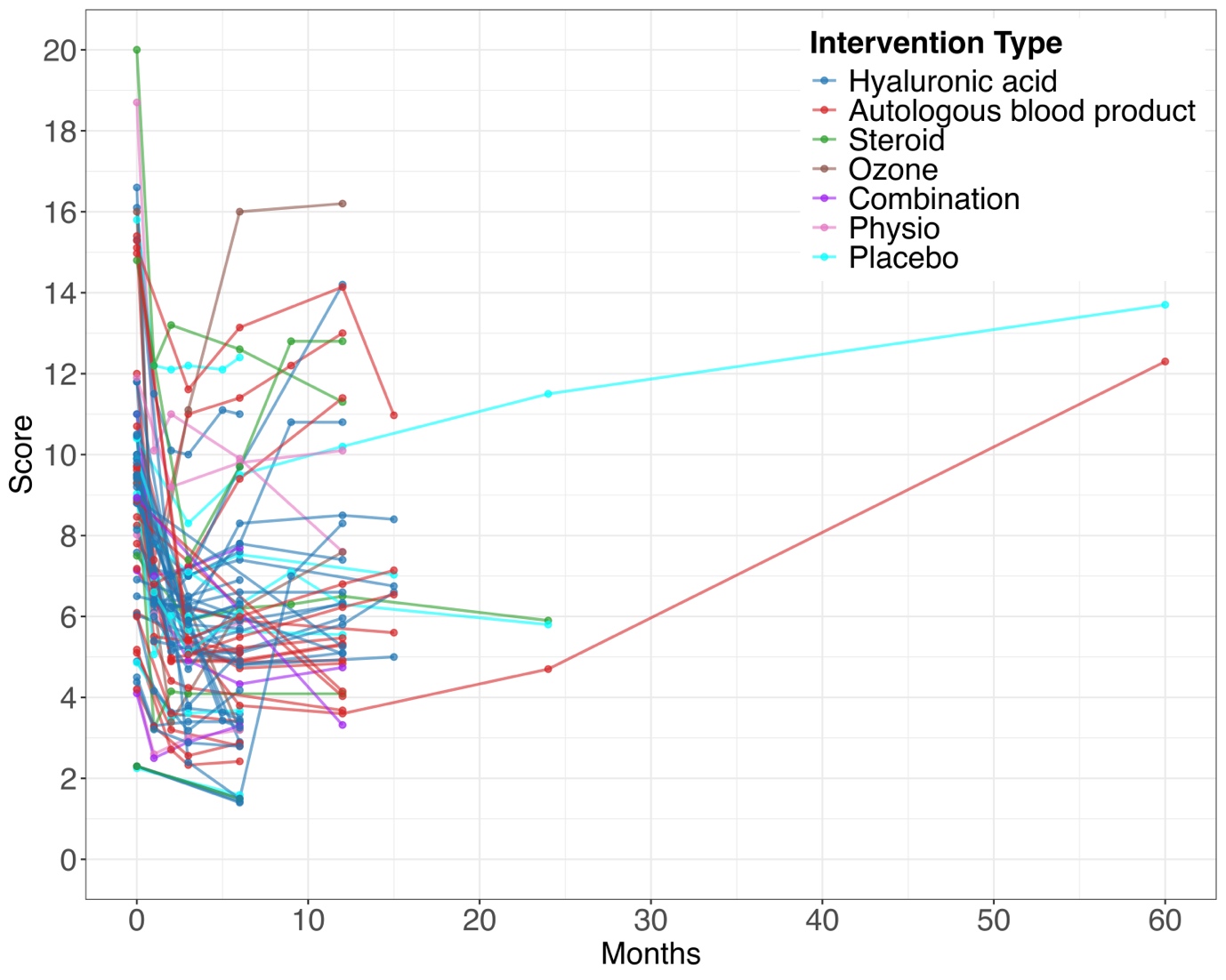


**Fig. 2.** Western Ontario and McMaster Universities Osteoarthritis Index (WOMAC) Pain scores separated by treatment type up to 60 months

**
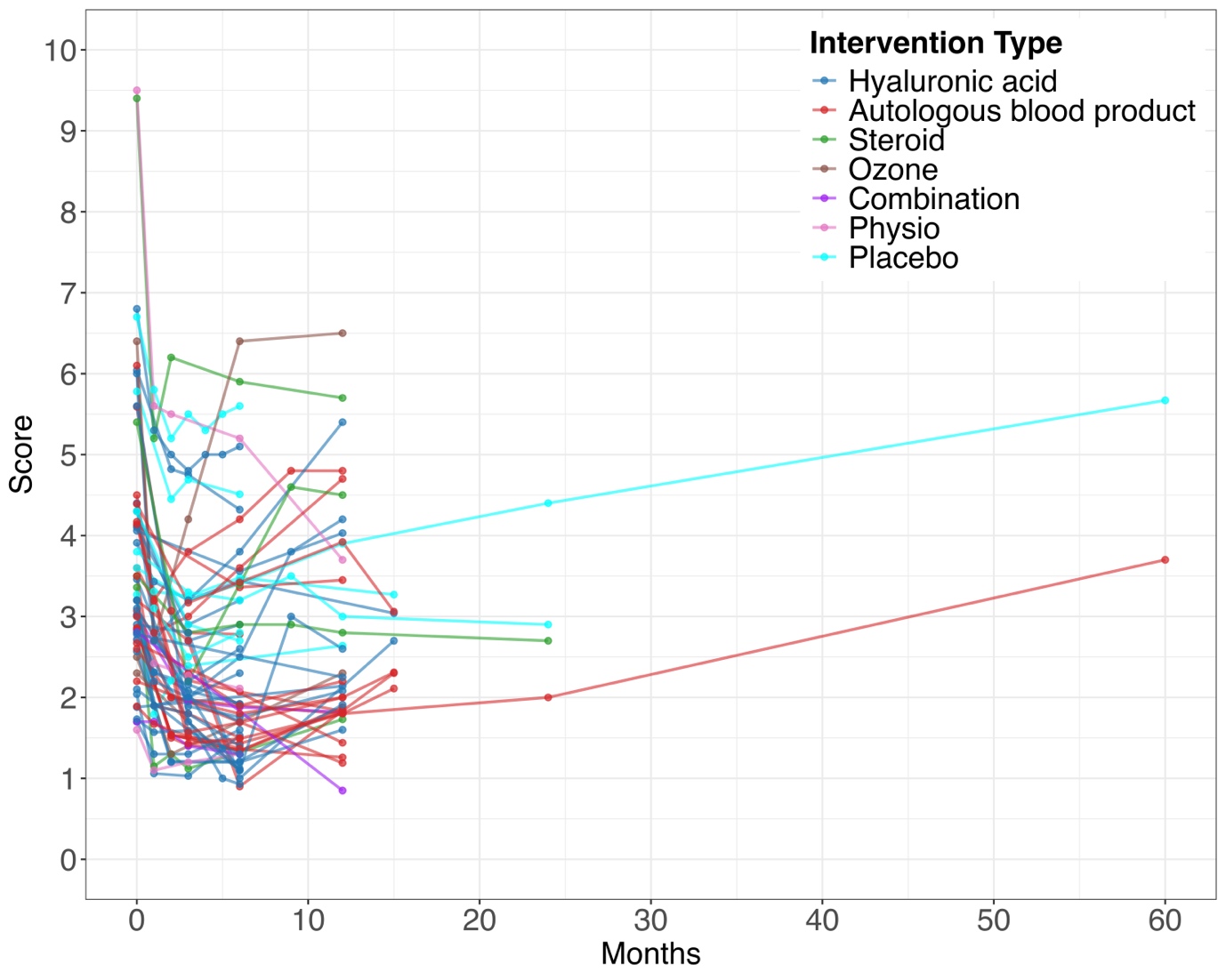
Fig. 3.** Western Ontario and McMaster Universities Osteoarthritis Index (WOMAC) Stiffness scores separated by treatment type up to 60 months


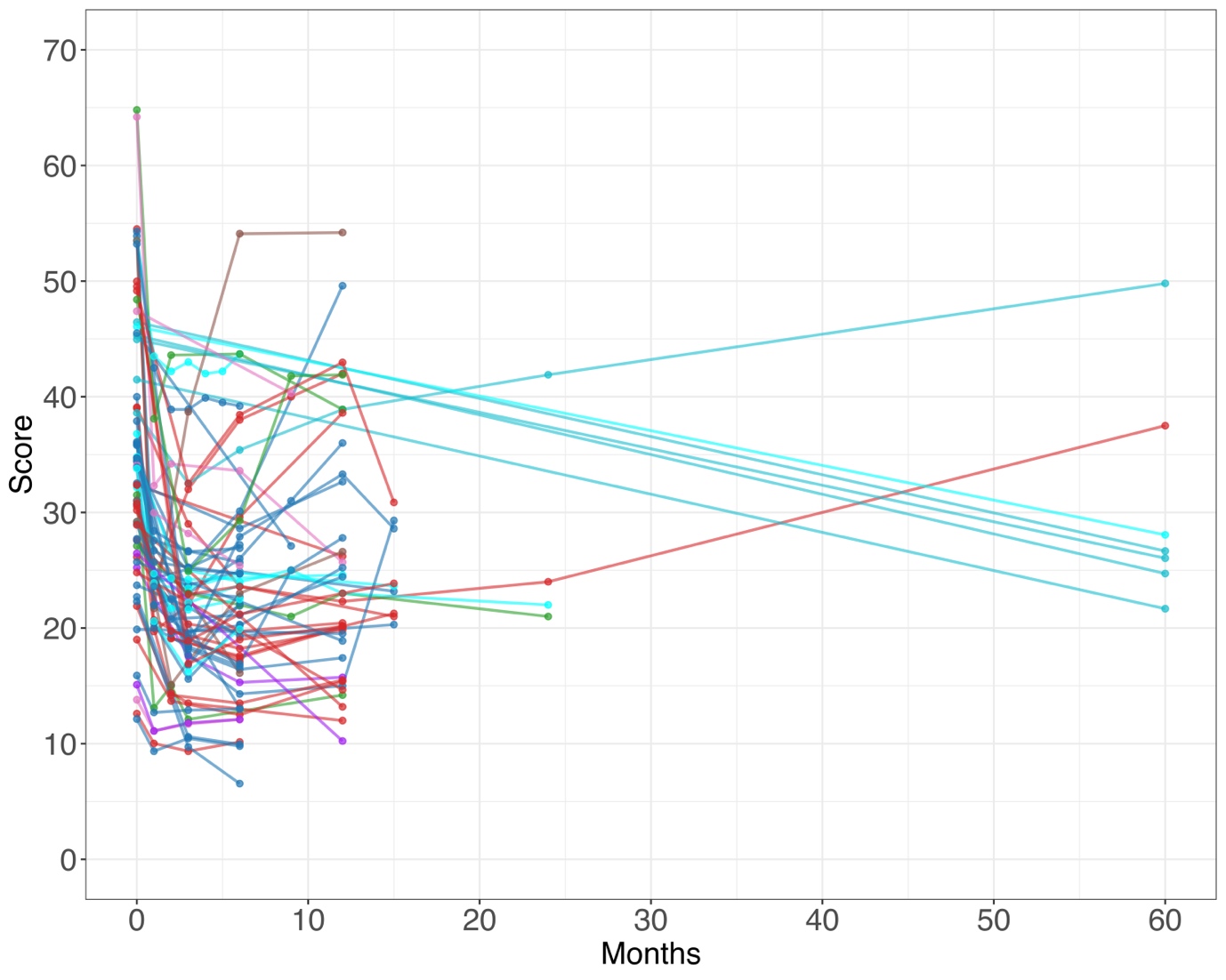

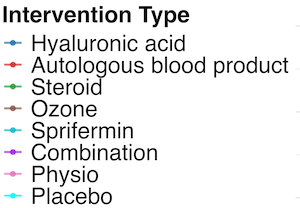


**Fig. 4.** Western Ontario and McMaster Universities Osteoarthritis Index (WOMAC) Physical Function (PF) scores separated by

m analysis

**TABLE S2A.**

Standardised mean change (SMC) scores with 95% confidence intervals (CI) for Western Ontario and McMaster Universities Osteoarthritis Index (WOMAC) at each time point overall according to the intervention received

| WOMAC-subscales | Time point (months) | Hyaluronic acid | Autologous blood product | Steroid | Adipose tissue product | Ozone | Sprifermin | Amniotic tissue product | Botox | Combination | Physio | Control |
| --- | --- | --- | --- | --- | --- | --- | --- | --- | --- | --- | --- | --- |
| Total | 1 | -2.85  (-4.79 to -0.91) | -2.71  (-4.2 to -1.23) | -0.93  (-2.07 to 0.21) | -0.14  (-2.1 to 1.82) | -3.77  (-6.1 to -1.45) | -0.18  (-1.16 to 0.8) | N/A | N/A | -1.85  (-3.28 to -0.42) | -1.05  (-3.03 to 0.92) | -0.05  (-1.24 to 1.13) |
|  | 3 | -3.61  (-5.56 to -1.65) | -2.19  (-3.17 to -1.22) | -3.38  (-6.72 to -0.05) | -0.28  (-2.24 to 1.69) | -1.92  (-3.99 to 0.14) | -0.36  (-1.34 to 0.62) | N/A | N/A | -1.3  (-2.45 to -0.15) | -1.05  (-3.02 to 0.92) | -0.11  (-1.5 to 1.28) |
|  | 6 | -3.01  (-4.47 to -1.55) | -1.78  (-2.55 to -1) | -2.44  (-4.58 to -0.3) | -0.42  (-2.38 to 1.55) | -1.09  (-3.36 to 1.18) | -0.54  (-1.52 to 0.45) | N/A | N/A | -0.66  (-1.79 to 0.48) | -1.04  (-3.01 to 0.93) | 0.11  (-1.28 to 1.49) |
|  | 9 | -3.78  (-8.08 to 0.51) | -1.64  (-5.4 to 2.13) | -2.64 (  -4.12 to -1.16) | -0.56  (-2.52 to 1.41) | -0.5  (-2.62 to 1.61) | -0.72  (-1.7 to 0.27) | N/A | N/A | -0.86  (-2.13 to 0.41) | -0.51  (-2.47 to 1.45) | 0.55  (-1.41 to 2.52) |
|  | 12 | \| -2.25  (-4.12 to -0.37) \| \| --- \| | -1.48  (-2.2 to -0.76) | -1.66  (-3.23 to -0.09) | -0.69  (-2.66 to 1.27) | 0.08 (-1.88 to 2.04) | -0.89  (-1.88 to 0.09) | N/A | N/A | -1.06  (-2.46 to 0.33) | -1.32  (-3.3 to 0.66) | 0.24  (-1.15 to 1.63) |
|  | 24 | 0.16  (-1.80 to 2.12) | -1.96  (-3.93 to 0.02) | N/A | -0.58  (-2.54 o 1.39) | N/A | -1.07  (-2.06 to -0.09) | N/A | N/A | N/A | -1.26  (-3.24 to 0.71) | 0.48  (-1.48 to 2.44) |
|  | 60 | 0.42  (-1.54 to 2.38) | 0.00  (-1.96 to 1.96) | N/A | -0.24  (-2.2 to 1.72) | N/A | -1.25  (-2.24 to -0.26) | N/A | N/A | N/A | -1.21 (-3.18 to 0.77) | 1.53  (-0.44 to 3.5) |
| Pain | 1 | -2.82  (-4.52 to -1.12) | -1.98  (-3.39 to -0.57) | -1.36  (-2.79 to 0.06) | N/A | -3.48  (-5.76 to -1.21) | N/A | N/A | N/A | -0.3  (-2.27 to 1.66) | -1.11  (-2.66 to 0.43) | -1.09  (-3.09 to 0.9) |
|  | 3 | -2.27  (-3.33 to -1.22) | -2.62  (-3.96 to -1.27) | -1.51  (-2.66 to -0.35) | N/A | -1.81  (-3.86 to 0.24) | N/A | N/A | N/A | -0.6  (-2.57 to 1.36) | -2.66  (-4.68 to -0.65) | -0.73  (-1.71 to 0.26) |
|  | 6 | -2.91  (-4.77 to -1.06) | -1.98  (-2.99 to -0.97) | -1.12  (-2.11 to -0.13) | N/A | -0.68  (-2.08 to 0.71) | N/A | N/A | N/A | -0.76  (-2.73 to 1.21) | -1.21  (-2.94 to 0.51) | -4.88  (-13.45 to 3.68) |
|  | 9 | -1.58 (-4.47 to 1.3) | 0.04  (-1.92 to 2) | -0.52  (-1.91 to 0.87) | N/A | -0.6  (-1.99 to 0.8) | N/A | N/A | N/A | -0.71  (-2.67 to 1.26) | -0.82  (-2.67 to 1.02) | -0.37  (-2.33 to 1.6) |
|  | 12 | -0.93 (-1.64 to -0.23) | -1.7  (-2.63 to -0.77) | -1.07  (-2.22 to 0.08) | N/A | -0.51  (-1.9 to 0.88) | N/A | N/A | N/A | -0.65  (-2.62 to 1.32) | -0.43 (-2.39 to 1.53) | -0.52  (-1.92 to 0.88) |
|  | 24 | N/A | -3.33  (-5.33 to -1.34) | -0.53  (-2.5 to 1.43) | N/A | N/A | N/A | N/A | N/A | N/A | N/A | -0.14  (-1.52 to 1.25) |
|  | 60 | N/A | 0.89  (-1.07 to 2.85) | N/A | N/A | N/A | N/A | N/A | N/A | N/A | N/A | 1.57  (-0.4 to 3.54) |

**TABLE S3B.**

Standardised mean change (SMC) scores with 95% confidence intervals (CI) for Western Ontario and McMaster Universities Osteoarthritis Index (WOMAC) at each time point overall according to the intervention received

| WOMAC-subscales | Time point (months) | Hyaluronic acid | Autologous blood product | Steroid | Adipose tissue product | Ozone | Sprifermin | Amniotic tissue product | Botox | Combination | Physio | Control |
| --- | --- | --- | --- | --- | --- | --- | --- | --- | --- | --- | --- | --- |
| Stiffness | 1 | -1.11  (-1.82 to -0.4) | -1.40  (-2.7 to -0.1) | -1.01  (-2.41 to 0.40) | N/A | -3.7  (-6.01 to -1.39) | N/A | N/A | N/A | -0.31  (-1.98 to 1.37) | -0.29  (-1.68 to 1.1) | -0.46 (-2.43 to 1.5) |
|  | 3 | -1.13  (-1.7 to -0.55) | -1.35  (-1.96 to -0.73) | -1.06  (-2.2 to 0.08) | N/A | -2.2  (-4.29 to -0.11) | N/A | N/A | N/A | -0.61 (-2.58 to 1.35) | -0.42  (-2.38 to 1.54) | -0.32  (-1.30 to 0.67) |
|  | 6 | -1.23  (-1.74 to -0.72) | -1.26  (-1.8 to -0.72) | -0.74  (-1.88 to 0.4) | N/A | -0.25  (-1.64 to 1.14) | N/A | N/A | N/A | -0.66  (-2.62 to 1.31) | -0.45  (-1.84 to 0.94) | -0.33  (-1.47 to 0.8) |
|  | 9 | -0.96  (-2.45 to 0.52) | 0.1  (-1.86 to 2.06) | -0.4  (-1.79 to 0.99) | N/A | -0.07  (-1.75 to 1.6) | N/A | N/A | N/A | -0.83 (-2.51 to 0.85) | -0.45 (-2.12 to 1.23) | -0.21  (-2.17 to 1.75) |
|  | 12 | \| -0.56  (-1.26 to 0.13) \| \| --- \| | -0.89  (-1.42 to -0.36) | -0.63  (-1.62 to 0.35) | N/A | 0.1  (-1.86 to 2.06) | N/A | N/A | N/A | -1.01 (-2.4 to 0.39) | -0.45 (-2.41 to 1.51) | -0.2 (-1.33 to 0.94) |
|  | 24 | N/A | -1 (-2.96 to 0.96) | -0.5  (-2.46 to 1.46) | N/A | N/A | N/A | N/A | N/A | N/A | N/A | -0.08 (-1.47 to 1.3) |
|  | 60 | N/A | 0.06 (-1.9 to 2.02) | N/A | N/A | N/A | N/A | N/A | N/A | N/A | N/A | 1.22 (-0.75 to 3.18) |
| Function | 1 | -3  (-5.11 to -0.88) | -2.19 (-3.84 to -0.53) | -0.91 (-2.41 to 0.58) | N/A | -3.66 (-5.96 to -1.35) | -0.16 (-1.14 to 0.82) | N/A | N/A | -0.19 (-1.86 to 1.48) | -0.73 (-2.11 to 0.66) | -1.02 (-2.42 to 0.38) |
|  | 3 | -3.14 (-5.12 to -1.16) | -1.78 (-2.57 to -0.99) | -2.32 (-4.92 to 0.27) | N/A | -1.7 (-3.74 to 0.34) | -0.32 (-1.3 to 0.67) | N/A | N/A | -0.38 (-2.34 to 1.58) | -1.56 (-3.54 to 0.42) | -0.82 (-1.8 to 0.17) |
|  | 6 | -2.67  (-4.13 to -1.21) | -1.34 (-1.93 to -0.75) | -1.5 (-3.88 to 0.88) | N/A | -0.89 (-2.79 to 1.01) | -0.47 (-1.45 to 0.51) | N/A | N/A | -0.5 (-2.46 to 1.47) | -1.31 (-3.23 to 0.6) | -0.69 (-1.82 to 0.45) |
|  | 9 | -2.87 (-6.86 to 1.13) | 0.08 (-1.88 to 2.04) | -0.97 (-2.36 to 0.43) | N/A | -0.4 (-2.33 to 1.53) | -0.63 (-1.61 to 0.35) | N/A | N/A | -0.74 (-2.42 to 0.93) | -0.41 (-2.37 to 1.55) | -0.44 (-2.4 to 1.52) |
|  | 12 | -1.33 (-2.75 to 0.08) | -1.13 (-1.68 to -0.57) | -0.86 (-1.85 to 0.13) | N/A | 0.08 (-1.88 to 2.04) | -0.79 (-1.77 to 0.2) | N/A | N/A | -0.99 (-2.39 to 0.4) | -0.44 (-2.4 to 1.53) | -0.52 (-1.65 to 0.62) |
|  | 24 | N/A | -1.57 (-3.54 to 0.39) | -0.56 (-2.53 to 1.4) | N/A | N/A | -0.95 (-1.93 to 0.04) | N/A | N/A | N/A | N/A | -0.22 (-1.61 to 1.17) |
|  | 60 | N/A | -0.17 (-2.13 to 1.79) | N/A | N/A | N/A | -1.1 (-2.09 to -0.12) | N/A | N/A | N/A | N/A | 0.01 (-2.03 to 2.06) |

**TABLE S4**

**Correlation matrix of the Western Ontario and McMaster Universities Osteoarthritis Index (WOMAC) Total scores at the different time points with 95% confidence intervals (CI) and number of studies**

|  | Baseline | 1 | 3 | 6 | 9 | 12 | 24 | 60 |
| --- | --- | --- | --- | --- | --- | --- | --- | --- |
| Baseline | 1 | 0.93  (0.84 to 0.97)  n=22 | 0.81  (0.67 to 0.90)  n=39 | 0.91  (0.84 to 0.95)  n=50 | 0.95  (0.80 to 0.99)  n=11 | 0.90  (0.80 to 0.95)  n=36 | 0.90  (-0.44 to 1.00)  n=4 | 0.81  (0.32 to 0.96)  n=6 |
| 1 | - | 1 | 0.96  (0.91 to 0.99)  n=20 | 0.97  (0.93 to 0.99)  n=22 | N/A | 0.91  (0.51 to 0.99)  n=7 | N/A | N/A |
| 3 | - | - | 1 | 0.90  (0.82 to 0.95)  n=37 | 0.95  (0.75 to 0.99)  n=9 | 0.82  (0.61 to 0.93)  n=21 | N/A | N/A |
| 6 | - | - | - | 1 | 0.99  (0.97 to 1.00)  n=9 | 0.90  (0.79 to 0.95)  n=27 | N/A | N/A |
| 9 |  | - |  |  | 1 | 0.96  (0.79 to 0.99)  n=8 | N/A | N/A |
| 12 | - | - | - | - | - | 1 | 1.00 (  0.87 to 1.00)  n=4 | 0.96 (  -0.02 to 1.00)  n=4 |
| 24 | - | - | - | - | - | - | 1 | 0.98  (0.25 to 1.00)  n=4 |
| 60 | - | - | - | - | - | - | - | 1 |

N/A = not applicable – inadequate studies to perform analysis

**TABLE S5**

Correlation matrix of the Western Ontario and McMaster Universities Osteoarthritis Index (WOMAC) Pain scores at the different time points with 95% confidence intervals (CI) and number of studies

|  | Baseline | 1 | 3 | 6 | 9 | 12 | 24 | 60 |
| --- | --- | --- | --- | --- | --- | --- | --- | --- |
| Baseline | 1 | 0.80  (0.53 to 0.92)  n=18 | 0.80  (0.63 to 0.90)  n=35 | 0.80  (0.67 to 0.88)  n=47 | 0.75  (-0.16 to 0.97)  n=6 | 0.58  (0.28 to 0.77)  n=31 | -0.42  (-0.98 to 0.91)  n=4 | N/A |
| 1 | - | 1 | 0.94  (0.83 to 0.98)  n=16 | 0.98  (0.93 to 0.99)  n=15 | N/A | 0.86  (0.50 to 0.97)  n=10 | N/A | N/A |
| 3 | - | - | 1 | 0.86  (0.74 to 0.93)  n=32 | 0.97  (0.71 to 1.00)  n=6 | 0.79  (0.511 to 0.92)  n=18 | 0.23  (-0.94 to 0.98)  n=4 | N/A |
| 6 | - | - | - | 1 | 0.94  (0.54 to 1.00)  n=6 | 0.84  (0.64 to 0.92)  n=23 | 0.11  (-0.95 to 0.97)  n=4 | N/A |
| 9 |  | - |  |  | 1 | 0.81  (-0.69 to 1.00)  n=4 | N/A | N/A |
| 12 | - | - | - | - | - | 1 | N/A | N/A |
| 24 | - | - | - | - | - | - | 1 | N/A |
| 60 | - | - | - | - | - | - | - | 1 |

N/A = not applicable – inadequate studies to perform analysis

**TABLE S6**

Correlation matrix of the Western Ontario and McMaster Universities Osteoarthritis Index (WOMAC) Stiffness scores at the different time points with 95% confidence intervals (CI) and number of studies

|  | Baseline | 1 | 3 | 6 | 9 | 12 | 24 | 60 |
| --- | --- | --- | --- | --- | --- | --- | --- | --- |
| Baseline | 1 | 0.82  (0.58 to 0.93)  n=18 | 0.85  (0.74 to 0.93)  n=33 | 0.77  (0.60 to 0.87)  n=40 | 0.54  (-0.49 to 0.94)  n=6 | 0.66  (0.42 to 0.82)  n=33 | -0.57  (-0.99 to 0.87)  n=4 | N/A |
| 1 | - | 1 | 0.96  (0.90 to 0.99)  n=16 | 0.97  (0.93 to 0.99)  n=15 | N/A | 0.87  (0.53 to 0.97)  n=10 | N/A | N/A |
| 3 | - | - | 1 | 0.902  (0.83 to 0.96)  n=30 | 0.41  (-0.60 to 0.92)  n=6 | 0.71  (0.38 to 0.87)  n=20 | 0.70  (-0.80 to 1.00)  n=4 | 0.96  (0.67 to 1.00)  n=6 |
| 6 | - | - | - | 1 | 0.96  (0.67 to 1.00)  n=6 | 0.96  (0.89 to 0.98)  n=25 | 0.74  (-0.77 to 1.00)  n=4 | N/A |
| 9 |  | - |  |  | 1 | 0.92  (0.45 to 1.00)  n=6 | N/A | N/A |
| 12 | - | - | - | - | - | 1 | 0.74  (-0.77 to 1.00)  n=4 | N/A |
| 24 | - | - | - | - | - | - | 1 | N/A |
| 60 | - | - | - | - | - | - | - | 1 |

N/A = not applicable – inadequate studies to perform analysis

**TABLE S7**

Correlation matrix of the Western Ontario and McMaster Universities Osteoarthritis Index (WOMAC) Physical Function (PF) scores at the different time points with 95% confidence intervals (CI) and number of studies

|  | Baseline | 1 | 3 | 6 | 9 | 12 | 24 | 60 |
| --- | --- | --- | --- | --- | --- | --- | --- | --- |
| Baseline | 1 | 0.79  (0.53 to 0.91)  n=16 | 0.71  (0.48 to 0.85)  n=32 | 0.879  (0.62 to 0.88)  n=38 | 0.87  (0.41 to 0.98)  n=8 | 0.68  (0.43 to 0.83)  n=32 | -0.02  (-0.96 to 0.96)  n=4 | 0.84  (0.24 to 0.98)  n=7 |
| 1 | - | 1 | 0.88  (0.70 to 0.995)  n=18 | 0.98  (0.94 to 0.99)  n=17 | N/A | 0.90  (0.57 to 0.98)  n=9 | N/A | N/A |
| 3 | - | - | 1 | 0.91  (0.81 to 0.96)  n=29 | 0.25  (-0.71 to 0.88)  n=6 | 0.42  (-0.04 to 0.74)  n=19 | 0.98  (0.37 to 1.00)  n=4 | N/A |
| 6 | - | - | - | 1 | 0.97  (0.77 to 1.00)  n=6 | 0.72  (0.45 to 0.87)  n=24 | 0.99  (0.57 to 1.00)  n=4 | N/A |
| 9 |  | - |  |  | 1 | 0.93  (0.48 to 0.99)  n=6 | 0.93  (0.48 to 0.99)  n=6 | N/A |
| 12 | - | - | - | - | - | 1 | 0.53  (-0.88 to 0.98)  n=4 | N/A |
| 24 | - | - | - | - | - | - | 1 | N/A |
| 60 | - | - | - | - | - | - | - | 1 |

N/A = not applicable – inadequate studies to perform analysis


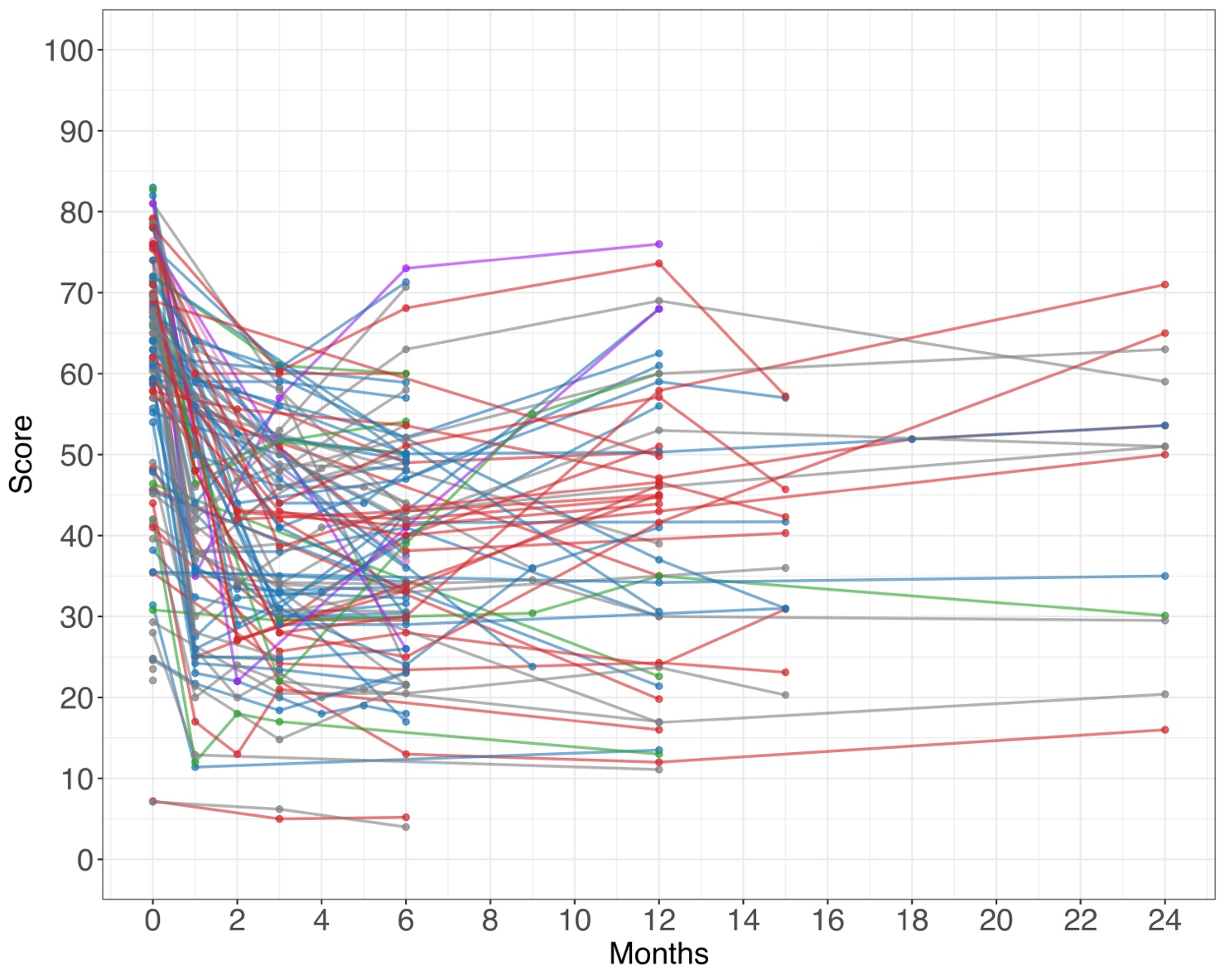


**Fig. 4.** Visual Analogues cores separated by treatment type up to 24 months

**TABLE S8.**

| Time point (months) | Hyaluronic acid | Autologous blood product | Steroid | Adipose tissue product | Ozone | Sprifermin | Amniotic tissue product | Botox | Combination | Physio | Placebo |
| --- | --- | --- | --- | --- | --- | --- | --- | --- | --- | --- | --- |
| 1 | -6.26  (-10.42 to -2.1) | -2.92  (-5.18 to -0.66) | -1.53  (-2.69 to -0.37) | -0.31  (-1.82 to 1.2) | -3.36  (-5.62 to -1.11) | N/A | N/A | -2.56  (-4.73 to -0.4) | -1.93  (-3.37 to -0.50) | -1.25  (-2.15 to -0.35) | -3.09  (-5.34 to -0.84) |
| 3 | -5.42  (-8.74 to -2.1) | -4.02  (-6.74 to -1.29) | -1.62  (-2.97 to -0.27) | -0.62  (-2.25 to 1.01) | -1.36  (-3.37 to 0.65) | N/A | N/A | -2.56  (-4.73 to -0.4) | -1.41  (-2.82 to 0.00) | -1.31  (-1.97 to -0.64) | -3.00  (-5.23 to -0.77) |
| 6 | -4.63  (-7.14 to -2.13) | -3.1 (-4.67 to -1.53) | -1.39 (-2.44 to -0.34) | -0.93 (-2.69 to 0.82) | -0.83 (-2.67 to 1) | N/A | N/A | N/A | -0.44  (-1.83 to 0.95) | -1.15  (-1.81 to -0.49) | N/A |
| 9 | -3.42 (-4.87 to -1.96) | -2.67 (-3.94 to -1.41) | -0.54 (-1.93 to 0.85) | -1.24 (-3.12 to 0.63) | -0.24 (-2.14 to 1.67) | N/A | N/A | N/A | N/A | -0.04  (-2.00 to 1.92) | -1.81  (-3.78 to 0.17) |
| 12 | -2.14 (-3.45 to -0.83) | -2.24 (-3.2 to -1.28) | -0.48 (-1.62 to 0.66) | -1.55 (-3.56 to 0.45) | 0.36 (-1.6 to 2.33) | N/A | N/A | N/A | N/A | N/A | N/A |
| 24 | -0.05 (-2.01 to 1.91) | -3.59 (-5.59 to -1.59) | -0.03 (-1.99 to 1.93) | -1.32 (-3.31 to 0.67) | N/A | N/A | N/A | N/A | N/A | -0.04  (-1.43 to 1.34) | N/A |
| 60 | 0.44 (-1.52 to 2.4) | 0.08 (-1.88 to 2.04) | N/A | -0.75 (-2.72 to 1.22) | N/A | N/A | N/A | N/A | N/A | 1.18  (-0.78 to 3.15) | N/A |

Standardised mean change (SMC) scores with 95% confidence intervals (CI) for Visual Analogue Scale (VAS) for pain at each time point according to the intervention received

**Fig. 5.** Knee Injury and Osteoarthritis Score (KOOS) sub-scales scores up to 12 months


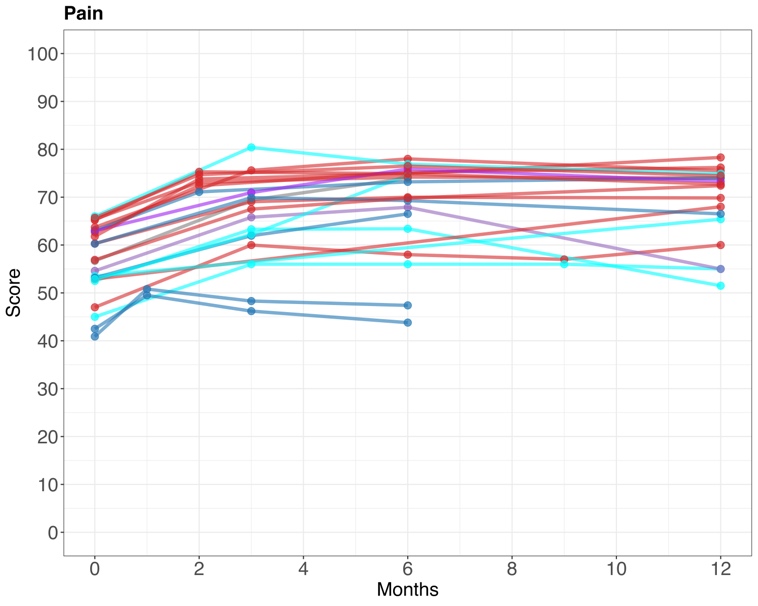

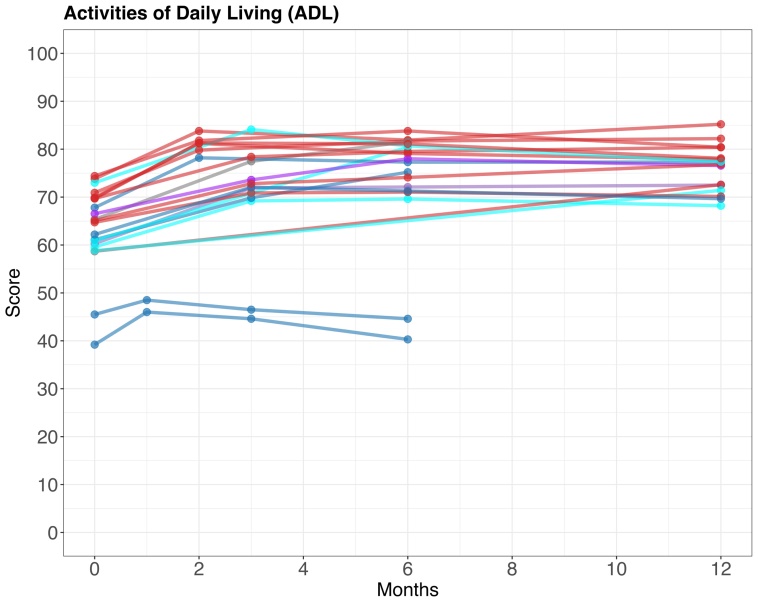

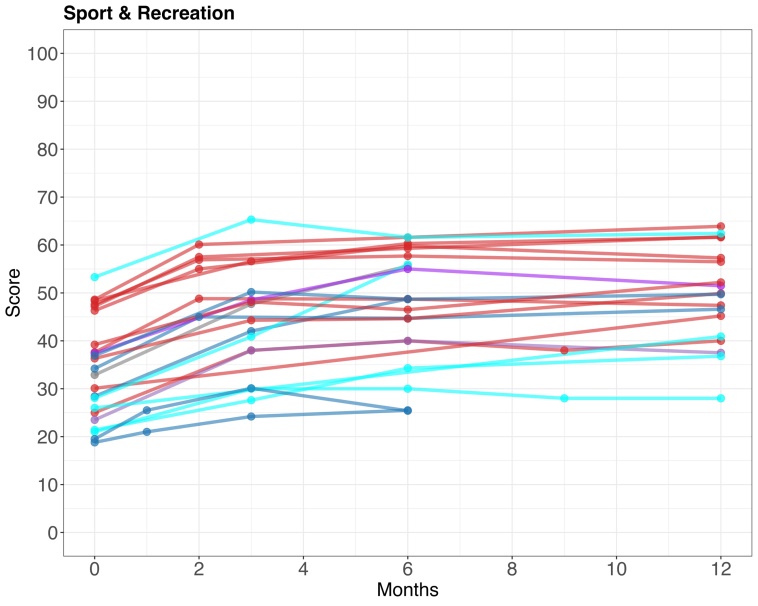

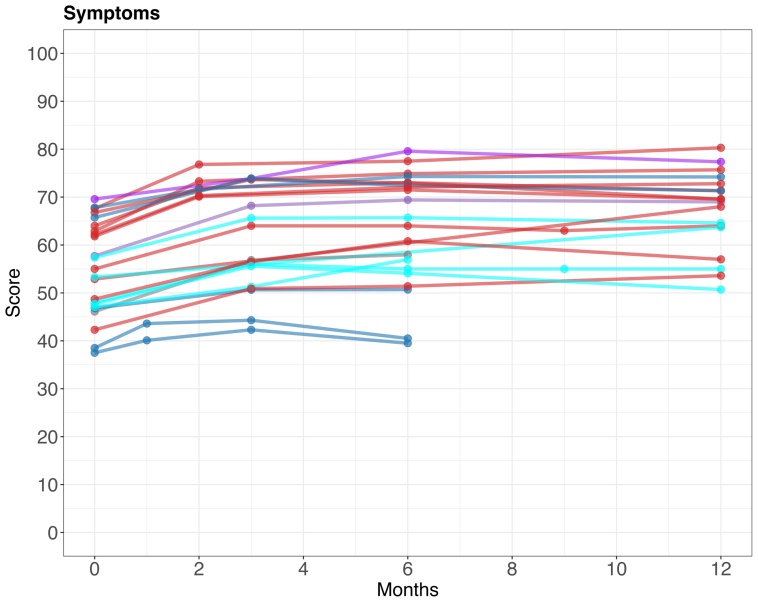

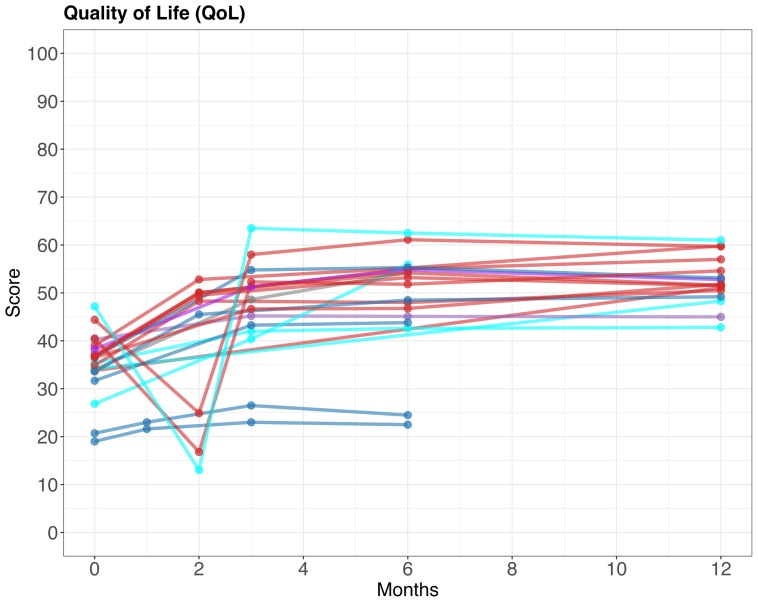

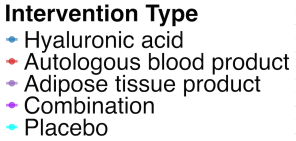

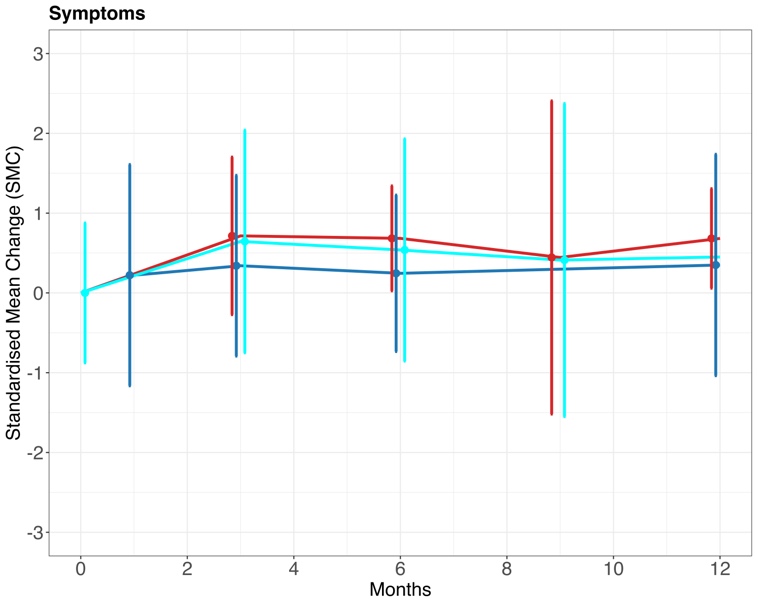

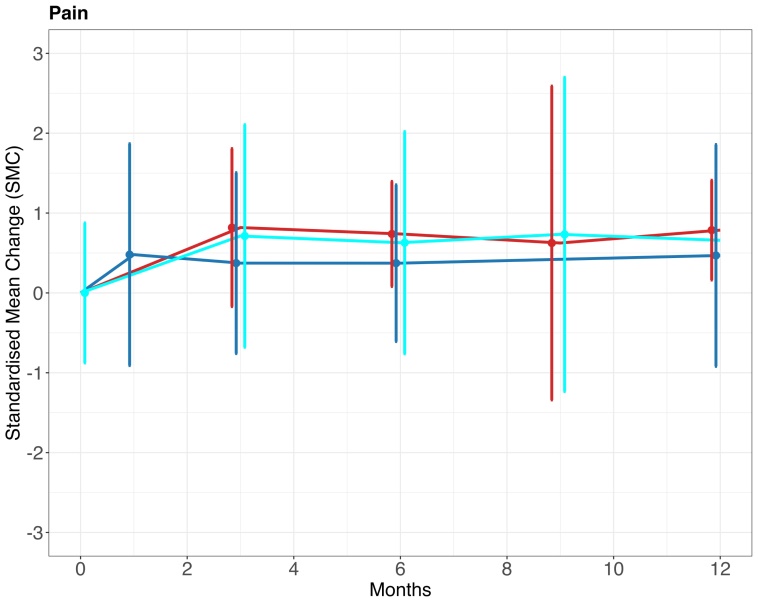

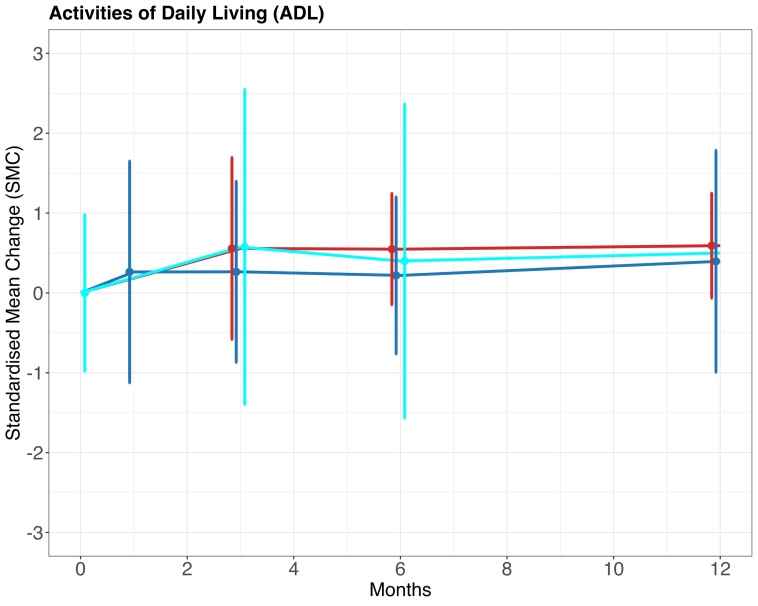

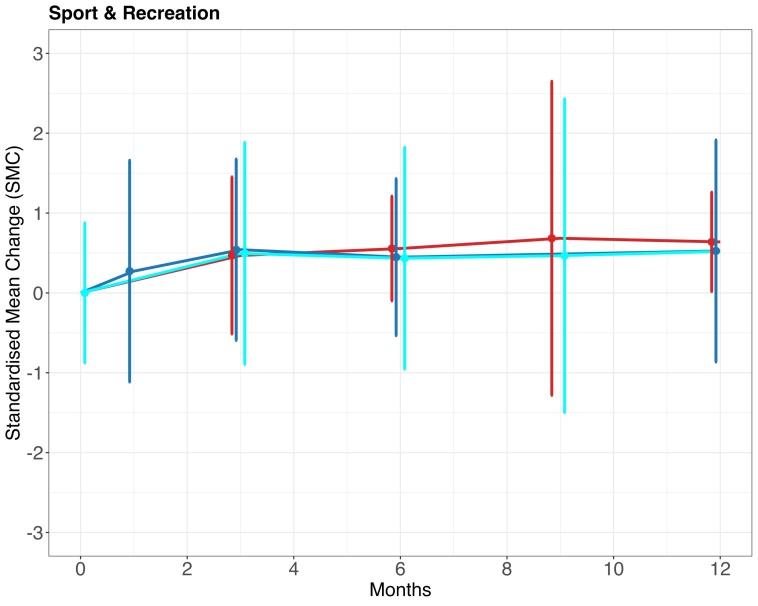

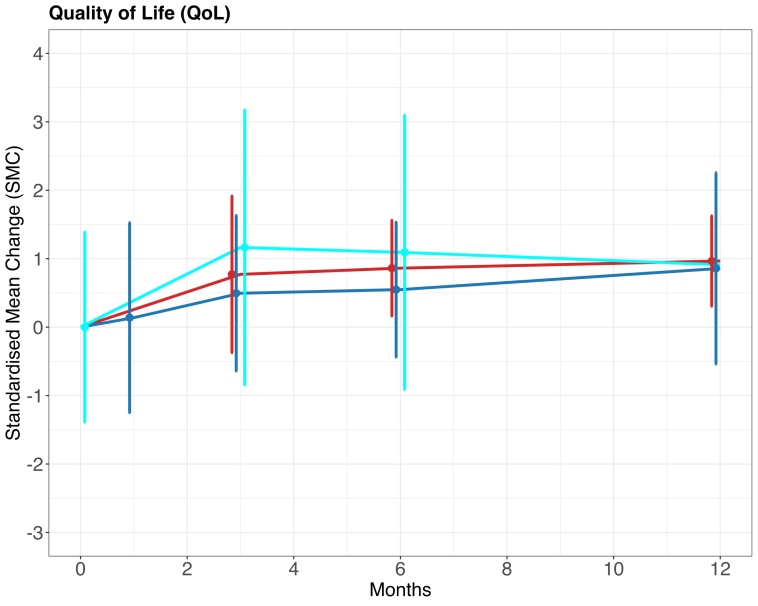


**Fig. 6.** Standardised mean change (SMC) scores for Knee Osteoarthritis Outcome Score (KOOS) for pain until 60 months (Blue=hyaluronic acid, red=autologous blood product, green=steroid, pink=physio, cyan=placebo)

**TABLE 9A.**

Standardised mean change (SMC) scores with 95% confidence intervals (CI) for Knee Injury and Osteoarthritis Score (KOOS) for pain at each time point according to the intervention received

| KOOS-subscales | Time point (months) | Hyaluronic acid | Autologous blood product | Combination | Placebo |
| --- | --- | --- | --- | --- | --- |
| Pain | 1 | 0.48  (-0.91 to 1.87) | N/A | N/A | N/A |
|  | 3 | 0.37 (-0.76 to 1.51) | 0.82 (-0.17 to 1.81) | 0.44 (-1.53 to 2.40) | 0.71 (-0.68 to 2.11) |
|  | 6 | 0.37 (-0.61 to 1.35) | 0.74 (0.08 to 1.4) | 0.7 (-1.26 to 2.67) | 0.63  (-0.76 to 2.02) |
|  | 9 | N/A | 0.63 (-1.34 to 2.59) | N/A | 0.73  (-1.23 to 2.70) |
|  | 12 | 0.47 (-0.92 to 1.86) | 0.79 (0.16 to 1.41) | 0.56 (-1.4 to 2.53) | N/A |
| Symptoms | 1 | 0.22 (-1.16 to 1.61) | N/A | N/A | N/A |
|  | 3 | 0.34 (-0.79 to 1.47) | 0.71 (-0.27 to 1.7) | 0.27 (-1.69 to 2.23) | 0.64  (-0.75 to 2.04) |
|  | 6 | 0.25 (-0.73 to 1.23) | 0.68 (0.03 to 1.34) | 0.63 (-1.33 to 2.6) | 0.54  (-0.85 to 1.93) |
|  | 9 | N/A | 0.44 (-1.52 to 2.41) | N/A | 0.41  (-1.55 to 2.37) |
|  | 12 | 0.35 (-1.04 to 1.74) | 0.68 (0.06 to 1.31) | 0.49 (-1.47 to 2.46) | N/A |
| Activities of daily living (ADL) | 1 | 0.26 (-1.12 to 1.65) | N/A | N/A | N/A |
|  | 3 | 0.26 (-0.87 to 1.4) | 0.56 (-0.58 to 1.69) | N/A | 0.58  (-1.40 to 2.55) |
|  | 6 | 0.22 (-0.76 to 1.2) | 0.55 (-0.15 to 1.24) | N/A | 0.40  (-1.57 to 2.36) |
|  | 9 | N/A | N/A | N/A | N/A |
|  | 12 | 0.4 (-0.99 to 1.78) | 0.59 (-0.06 to 1.25) | N/A | 0.50  (-0.89 to 1.89) |

**TABLE 9B.**

Standardised mean change (SMC) scores with 95% confidence intervals (CI) for Knee Injury and Osteoarthritis Score (KOOS) for pain at each time point according to the intervention received

| KOOS-subscales | Time point (months) | Overall | Hyaluronic acid | Autologous blood product | Combination | Placebo |
| --- | --- | --- | --- | --- | --- | --- |
| Sport & Recreation | 1 | 0.39  (-1.58 to 2.35) | 0.27 (-1.11 to 1.66) | N/A | N/A | N/A |
|  | 3 | 0.57  (-0.31 to 1.45) | 0.54 (-0.59 to 1.68) | 0.47 (-0.51 to 1.45) | 0.43 (-1.53 to 2.39) | 0.49  (-0.89 to 1.88) |
|  | 6 | 0.49  (-0.20 to 1.19) | 0.45 (-0.53 to 1.43) | 0.56 (-0.1 to 1.21) | 0.68 (-1.29 to 2.65) | 0.43  (-0.95 to 1.82) |
|  | 9 | 0.58  (-0.81 to 1.96) | N/A | 0.68 (-1.28 to 2.65) | N/A | 0.47  (-1.50 to 2.43) |
|  | 12 | 0.61  (-0.05 to 1.27) | 0.53 (-0.86 to 1.91) | 0.64 (0.02 to 1.26) | 0.55 (-1.42 to 2.51) | 0.52  (-0.61 to 1.65) |
| Quality of life (QoL) | 1 |  | 0.14 (-1.25 to 1.52) | N/A | N/A | N/A |
|  | 3 | 0.82  (-0.32 to 1.97) | 0.5 (-0.64 to 1.63) | 0.77 (-0.37 to 1.91) | 0.73 (-1.24 to 2.7) | 1.16  (-0.84 to 3.17) |
|  | 6 | 0.81  (0.00 to 1.62) | 0.55 (-0.44 to 1.53) | 0.86 (0.16 to 1.56) | 0.93 (-1.05 to 2.9) | 1.09  (-0.91 to 3.09) |
|  | 9 | N/A | N/A | N/A | N/A | N/A |
|  | 12 | 1.00  (0.25 to 1.75) | 0.86 (-0.54 to 2.25) | 0.97 (0.31 to 1.62) | 0.81 (-1.16 to 2.79) | 0.91  (-0.49 to 2.31) |


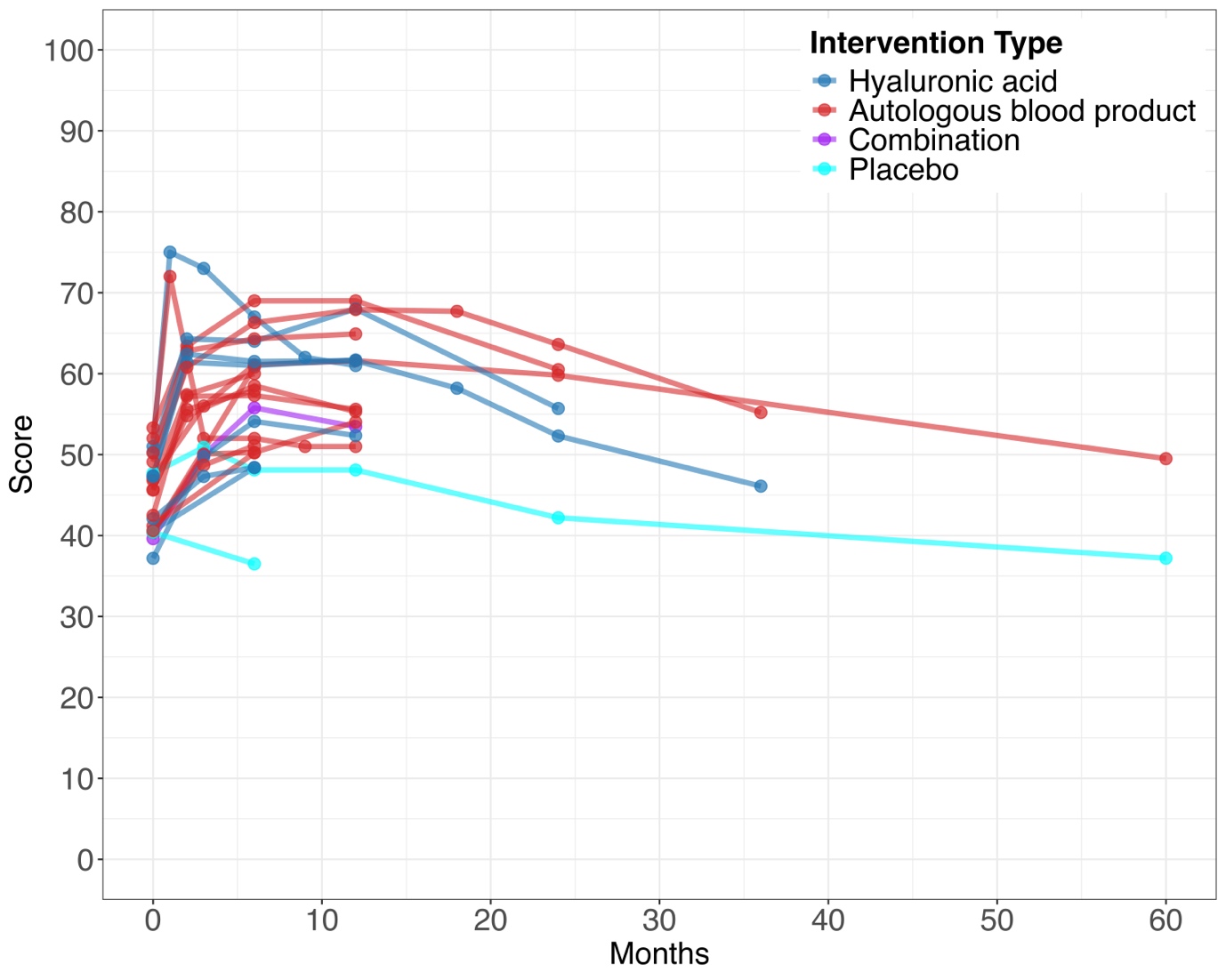


**Fig. 7.** International Knee Documentation Centre (IKDC) sub-scales scores up to 60 months

**
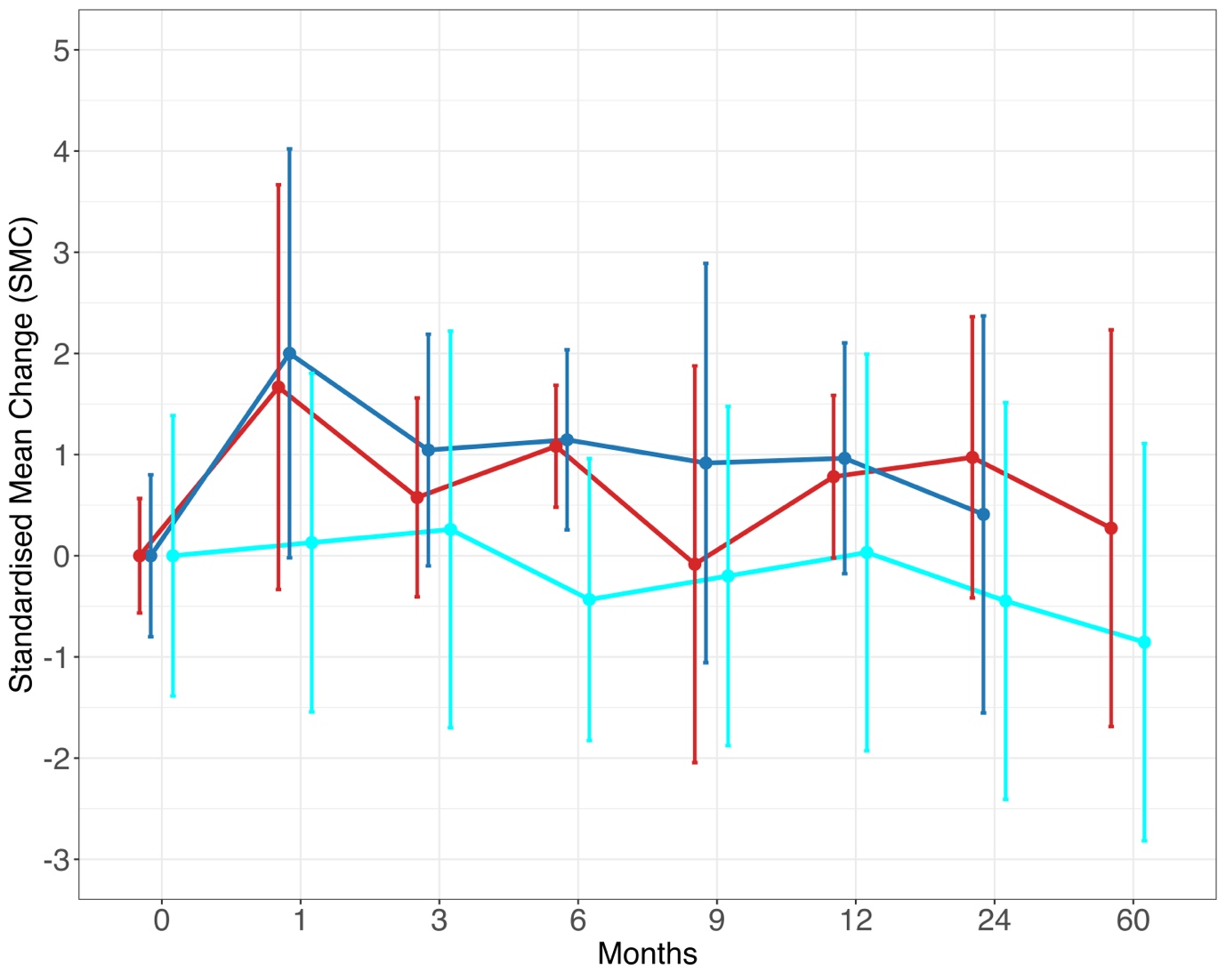
**

**Fig. 8.** Standardised mean change (SMC) scores for International Knee Documentation Centre (IKDC) until 60 months (Blue= control, Red=injection)

**TABLE 10.**

Standardised mean change (SMC) scores with 95% confidence intervals (CI) for International Knee Documentation Centre (IKDC) for pain at each time point according to the intervention received

| Time point (months) | Hyaluronic acid | Autologous blood product | Combination | Placebo |
| --- | --- | --- | --- | --- |
| 1 | 2 (-0.02 to 4.02) | 1.67 (-0.33 to 3.67) | 0.36 (-1.61 to 2.32) | 0.13 (-1.54 to 1.8) |
| 3 | 1.04 (-0.1 to 2.19) | 0.58 (-0.41 to 1.56) | 0.71 (-1.26 to 2.68) | 0.26  (-1.70 to 2.22) |
| 6 | 1.15 (0.26 to 2.04) | 1.08 (0.48 to 1.69) | 1.11 (-0.87 to 3.09) | -0.43  (-1.83 to 0.96) |
| 9 | 0.92 (-1.06 to 2.89) | -0.08 (-2.04 to 1.88) | 1.03 (-0.94 to 3.01) | -0.2 (-1.88 to 1.48) |
| 12 | 0.96 (-0.18 to 2.1) | 0.78 (-0.02 to 1.59) | 0.95 (-1.02 to 2.93) | 0.03  (-1.93 to 1.99) |
| 24 | 0.41 (-1.55 to 2.37) | 0.97 (-0.42 to 2.36) | N/A | -0.45  (-2.41 to 1.51) |
| 60 | N/A | 0.27 (-1.69 to 2.23) | N/A | -0.85  (-2.82 to 1.11) |

**Fig. 9**
Risk of bias for all included studies as via the Risk of Bias (RoB) 2.0 tool

| **Study ID** | **D1** | **D2** | **D3** | **D4** | **D5** | **Overall** |  |  |  |
| --- | --- | --- | --- | --- | --- | --- | --- | --- | --- |
| Al-Omran 2014 |  |  |  |  |  |  |  |  | Low risk |
| Altman 1998 |  |  |  |  |  |  |  |  | Some concerns |
| Altman 2004 |  |  |  |  |  |  |  |  | High risk |
| Auw Yang 2008 |  |  |  |  |  |  |  |  |  |
| Baltzer 2009 |  |  |  |  |  |  |  | D1 | Randomisation process |
| Bansal 2021 |  |  |  |  |  |  |  | D2 | Deviations from the intended interventions |
| Barfod 2025 |  |  |  |  |  |  |  | D3 | Missing outcome data |
| Bennell 2021 |  |  |  |  |  |  |  | D4 | Measurement of the outcome |
| Berenbaum 2011 |  |  |  |  |  |  |  | D5 | Selection of the reported result |
| Brandt 2001 |  |  |  |  |  |  |  |  |  |
| Buendía‑López 2018 |  |  |  |  |  |  |  |  |  |
| Cerza 2012 |  |  |  |  |  |  |  |  |  |
| Chen 2020 |  |  |  |  |  |  |  |  |  |
| Chevalier 2010 |  |  |  |  |  |  |  |  |  |
| Chu 2022 |  |  |  |  |  |  |  |  |  |
| Conrozier 2016 |  |  |  |  |  |  |  |  |  |
| Dara 2025 |  |  |  |  |  |  |  |  |  |
| de Campos 2013 |  |  |  |  |  |  |  |  |  |
| Deyle 2020 |  |  |  |  |  |  |  |  |  |
| Di Martino 2018 |  |  |  |  |  |  |  |  |  |
| Di Martino 2022 |  |  |  |  |  |  |  |  |  |
| Dougados 1993 |  |  |  |  |  |  |  |  |  |
| Duymus 2017 |  |  |  |  |  |  |  |  |  |
| Eckstein 2021 |  |  |  |  |  |  |  |  |  |
| Elawamy 2021 |  |  |  |  |  |  |  |  |  |
| Farr 2019 |  |  |  |  |  |  |  |  |  |
| Filardo 2012 |  |  |  |  |  |  |  |  |  |
| Fossati 2024 |  |  |  |  |  |  |  |  |  |
| Görmeli 2017 |  |  |  |  |  |  |  |  |  |
| Guo 2018 |  |  |  |  |  |  |  |  |  |
| Housman 2014 |  |  |  |  |  |  |  |  |  |
| Hsieh 2022 |  |  |  |  |  |  |  |  |  |
| Huang 2011 |  |  |  |  |  |  |  |  |  |
| Huang 2019 |  |  |  |  |  |  |  |  |  |
| Huskisson 1999 |  |  |  |  |  |  |  |  |  |
| Kapoor 2024 |  |  |  |  |  |  |  |  |  |
| Kahan 2003 |  |  |  |  |  |  |  |  |  |
| Karlsson 2002 |  |  |  |  |  |  |  |  |  |
| Lewis 2022 |  |  |  |  |  |  |  |  |  |
| Li 2024 |  |  |  |  |  |  |  |  |  |
| Lomonte 2015 |  |  |  |  |  |  |  |  |  |
| Lundsgaard 2008 |  |  |  |  |  |  |  |  |  |
| Maheu 2011 |  |  |  |  |  |  |  |  |  |
| McAlindon 2017 |  |  |  |  |  |  |  |  |  |
| Migliore 2021 |  |  |  |  |  |  |  |  |  |
| Nunes-Tamashiro 2022 |  |  |  |  |  |  |  |  |  |
| Park 2021 |  |  |  |  |  |  |  |  |  |
| Petterson 2019 |  |  |  |  |  |  |  |  |  |
| Raeissadat 2015 |  |  |  |  |  |  |  |  |  |
| Raeissadat 2018 |  |  |  |  |  |  |  |  |  |
| Raeissadat 2020 |  |  |  |  |  |  |  |  |  |
| Raeissadat 2021 |  |  |  |  |  |  |  |  |  |
| Raman 2008 |  |  |  |  |  |  |  |  |  |
| Raynauld 2005 |  |  |  |  |  |  |  |  |  |
| Rezasoltani 2020 |  |  |  |  |  |  |  |  |  |
| Romandini 2024 |  |  |  |  |  |  |  |  |  |
| Saccomanno 2015 |  |  |  |  |  |  |  |  |  |
| Sdeek 2021 |  |  |  |  |  |  |  |  |  |
| Shoma 2021 |  |  |  |  |  |  |  |  |  |
| Siddharth 2017 |  |  |  |  |  |  |  |  |  |
| Srikanth 2023 |  |  |  |  |  |  |  |  |  |
| Sun 2017 |  |  |  |  |  |  |  |  |  |
| Taftain 2021 |  |  |  |  |  |  |  |  |  |
| Trueba Davilio 2015 |  |  |  |  |  |  |  |  |  |
| van der Weegen 2015 |  |  |  |  |  |  |  |  |  |
| Wang 2018 |  |  |  |  |  |  |  |  |  |
| Wang 2022 |  |  |  |  |  |  |  |  |  |
| Yu 2018 |  |  |  |  |  |  |  |  |  |
| Yurtbay 2022 |  |  |  |  |  |  |  |  |  |
| Zaffagnini 2022 |  |  |  |  |  |  |  |  |  |
| Zhang 2015 |  |  |  |  |  |  |  |  |  |
| Zhang 2022 |  |  |  |  |  |  |  |  |  |
| Zhuang 2024 |  |  |  |  |  |  |  |  |  |
